# Body mass index and healthcare costs: using genetic variants from the HUNT study as instrumental variables

**DOI:** 10.1186/s12913-022-07597-z

**Published:** 2022-03-25

**Authors:** Christina Hansen Edwards, Gunnhild Åberge Vie, Jonas Minet Kinge

**Affiliations:** 1 Department of Public Health and Nursing, NTNU, Norwegian University of Science and Technology, Trondheim, Norway; 2 Centre for Fertility and Health & Centre for Disease Burden, Norwegian Institute of Public Health, Oslo, Norway; 3 Department of Health Management and Health Economics, Institute of Health and Society, University of Oslo, Oslo, Norway

**Keywords:** Body mass index, Health care costs, Causation, Mendelian randomization, Instrumental variables, Obesity

## Abstract

**Background:**

Past studies have found associations between obesity and healthcare costs, however, these studies have suffered from bias due to omitted variables, reverse causality, and measurement error.

**Methods:**

We used genetic variants related to body mass index (BMI) as instruments for BMI; thereby exploiting the natural randomization of genetic variants that occurs at conception. We used data on measured height and weight, genetic information, and sociodemographic factors from the Nord-Trøndelag Health Studies (HUNT), and individual-level registry data on healthcare costs, educational level, registration status, and biological relatives. We studied associations between BMI and general practitioner (GP)-, specialist-, and total healthcare costs in the Norwegian setting using instrumental variable (IV) regressions, and compared our findings with effect estimates from ordinary least squares (OLS) regressions. The sensitivity of our findings to underlying IV-assumptions was explored using two-sample Mendelian randomization methods, non-linear analyses, sex-, healthcare provider-, and age-specific analyses, within-family analyses, and outlier removal. We also conducted power calculations to assess the likelihood of detecting an effect given our sample 60,786 individuals.

**Results:**

We found that increased BMI resulted in significantly higher GP costs; however, the IV-based effect estimate was smaller than the OLS-based estimate. We found no evidence of an association between BMI and specialist or total healthcare costs.

**Conclusions:**

Elevated BMI leads to higher GP costs, and more studies are needed to understand the causal mechanisms between BMI and specialist costs.

**Supplementary Information:**

The online version contains supplementary material available at 10.1186/s12913-022-07597-z.

## Introduction

Obesity is highly prevalent in populations around the world [1], and a vast array of prevention strategies and treatments have been proposed to mitigate the epidemic [2–5]. Economic evaluations of potential strategies and treatments are needed to identify how resources should be invested. Correct identification of cost-effective interventions requires that accurate data on the costs of obesity are used in economic evaluations.

A multitude of studies have assessed the costs of obesity, and the findings of these studies have been summarized in several reviews [6–16] that conclude that the economic burden of obesity is substantial. Traditionally, studies have typically used cross-sectional or longitudinal data to assess the costs of obesity [14]. These study designs have some shortcomings that can lead to bias when estimating the effect of body mass index (BMI) on healthcare costs. One of the main challenges is that several obesity-associated diseases are both risk factors for obesity and complications of obesity, and that healthcare use in itself can result in increased BMI (for example due to side effects of medications [17]). Studies using cross-sectional data cannot overcome this issue because adjusting for obesity-related diseases could lead to over-adjustment. Data from longitudinal studies can be used to net out all time constant characteristics. However, bias from variables that change over time will still be present, which means that simultaneity bias can be reduced, but not eliminated by using longitudinal data.

Another limitation is that most past studies use self-reported BMI or healthcare costs in their calculations. Kent et al., 2017 [14] found that 55 of the 75 studies included in their review used self-reported weight and/or height to estimate BMI. Measurement error in BMI due to self-reporting bias typically leads to an underestimation of true BMI [18, 19]. Moreover 16 of the 75 studies used self-reported data on healthcare costs [14]. Often, individuals are unable to correctly recall the number of past healthcare contacts made, especially if contact frequency is high [20]. In standard linear regressions, measurement error in the independent variable may bias regression estimates towards zero, and measurement error in the dependent variable may increase variance hence decreasing power to detect a causal effect.

One way to overcome at least some of these endogeneity issues is by using an instrumental variable (IV) approach. In IV-analyses, omitted variable bias can be avoided if the instrument is uncorrelated with the omitted variables. In the context of obesity, simultaneity bias can also be largely avoided if the instrument used is constant throughout life. Measurement error can lead to bias even though an IV-approach is used [21], however, by using measured height and weight data and registry data on healthcare utilization, as we have done in the current study, we increase reliability of estimates from IV models.

In recent studies, the natural randomization that occurs due to the random allocation of genetic variants at conception has been exploited by using genetic information as instrumental variables. The first studies to make use of genetic information in IV-analyses used the height and/or weight of biological relative(s) as instruments [22–26]. These studies found that elevated BMI was associated with higher healthcare costs than when using non-IV methods. A potential limitation of using anthropometric measures of relatives is that effects of the household environment on the association between BMI and healthcare costs cannot be ruled out.

In two more recent studies, Dixon et al., 2020 [27], and Kurz and Laxy, 2020 [28] have tried to minimize bias from environmental concerns by conducting Mendelian randomization (MR) analyses, that is, by using genetic variants associated with BMI as instruments. The first study used a UK Biobank sample, and found that obesity was associated with higher hospital costs than when using non-IV models [27]. The second study, using data from South-Western Germany, found that obesity resulted in total healthcare costs that were over twice as large compared with non-IV based estimates [28]. Both of these studies have important limitations. The sample used by Kurz and Laxy, 2020 [28] is small for the MR context (*n* = 2796), and the majority (99.5%) of the individuals included in the sample used by Dixon et al., 2020 [27] were between 40 and 69 years of age. In addition, as Dixon et al., 2020 [27] point out, their sample population has lower mortality, lower levels of morbidity increasing behaviors, and are better educated than the wider UK population. Moreover, neither of these studies investigated the effect of BMI on general practitioner (GP) costs. Studying the effects of GP costs gives an increased understanding of the consequences of obesity, because the mechanisms driving GP costs may differ from those of specialist costs.

Our study uses a sample with an appropriate size for the MR context, includes adults aged 20 years and above, and can be considered largely representative of the Norwegian population. We have combined genotyped data from biological samples with data on measured height and weight, registry-based healthcare costs, sociodemographic variables, and relatedness. The objective of the study was to use genetic variants associated with BMI as instruments to estimate the effect of BMI on GP-, specialist-, and total costs in Norway. This was achieved by: 1) Estimating associations between BMI and GP-, specialist-, and total costs using ordinary-least squares (OLS) regression. 2) Re-estimating the same associations using two-stage least squares (2SLS) with three different genetic instruments that were generated based on 97 BMI-associated genetic variants [29]. 3) Conducting sensitivity analyses, including two-sample methods (inverse-variance weighted, MR-Egger, median-weighted, and modal weighted estimation), non-linear methods, sex-, healthcare provider, and age-specific analyses, within-family analyses using family-fixed effects, and outlier removal; to explore the robustness of our estimates and the validity of our instruments. We then discussed the findings from stages 1–3 and inferred about the causal effect of obesity on healthcare costs.

## Methods

### Data

**Fig. 1:**
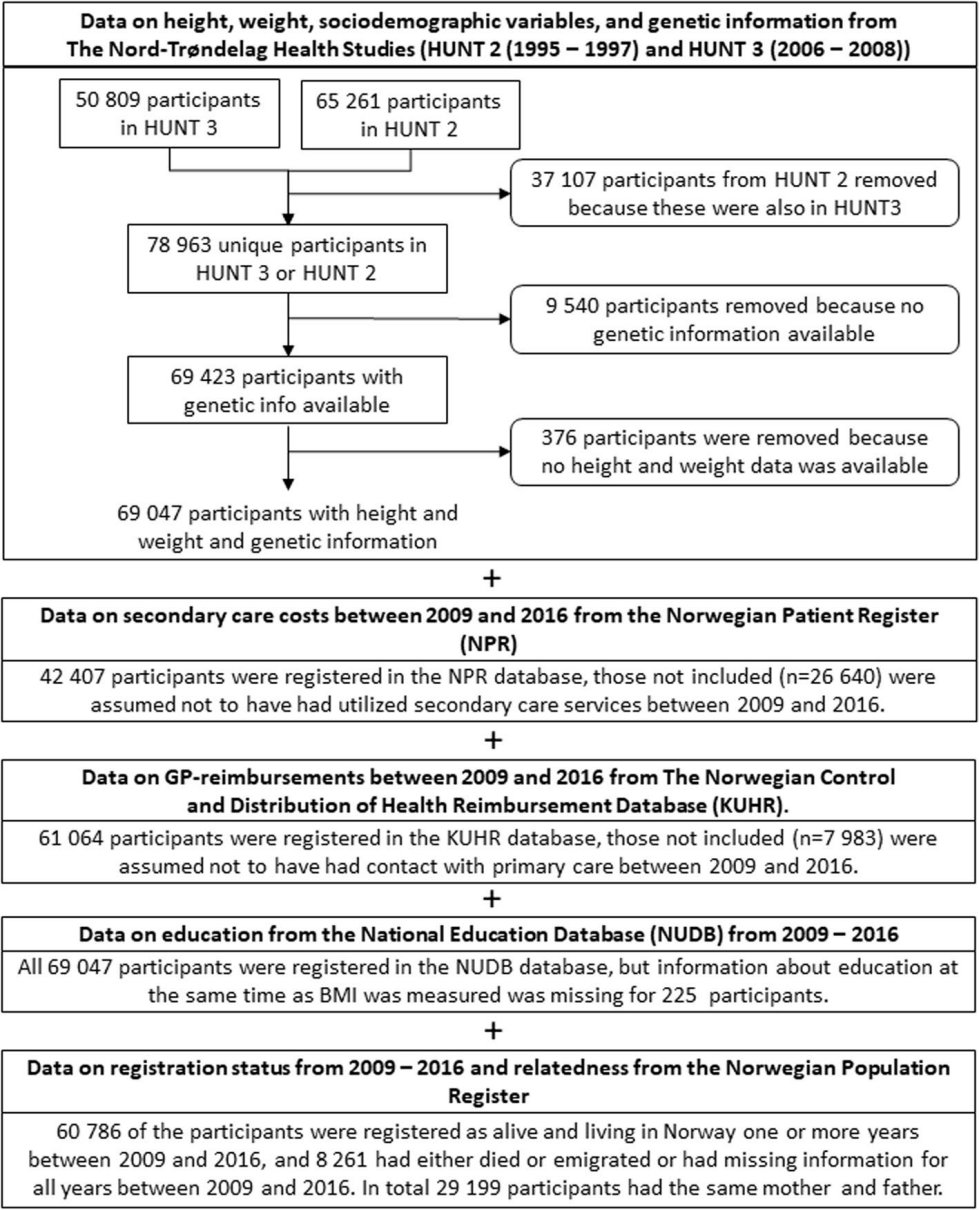
Overview of data sources used in the study

In this study, we used a combination of survey data and registry-based data. The five primary data sources used are presented in Fig. 1.

#### Data on BMI, sociodemographic factors, and genetic information

The HUNT study has been described in detail elsewhere [30, 31]. In brief, the HUNT study is a population-based cohort study. All inhabitants of Nord-Trøndelag (a rural geographical region in Central Norway) who turned 20 years of age during the study period were invited to participate. So far, four waves have been conducted, and we have used data from the second (HUNT 2) and third (HUNT 3) waves. The first wave did not contain genetic information, and the results from the fourth wave were unavailable at study initiation. Of those that were invited to take part in the study, 69.5% (*n* = 65,261) of adults in HUNT 2, and 54.1% (*n* = 50,809) of the adults in HUNT 3 participated. Data were collected between August 1995 and June 1997 (HUNT 2), and between October 2006 and June 2008 (HUNT 3). The studies consisted of questionnaires, clinical measurements, and collection of biological materials for DNA genotyping (performed using Illumina HumanCoreExome arrays). We used information about participants’ measured height (clinically measured to the nearest centimeter) and weight (clinically measured to the nearest half-kilogram without shoes and while participants were wearing light clothing) [30], socioeconomic variables (including age, sex, marital status, urbanity, and smoking status), genetic variants related to obesity, and genetic principal components. A description of the handling of the genotyped data, including information about the quality control and imputation procedures is included in the Additional Information File.

If the same individuals had participated in both HUNT 2 and HUNT 3, we used data from HUNT 3 (Fig. 1) since this was closer to the time at which healthcare costs were measured.

#### Data on healthcare costs

The Norwegian healthcare system is divided into primary- and specialist care. All registered inhabitants have a right to a regular GP, and approximately 99% of inhabitants are registered within the GP system [32]. GP services are part of the primary care sector, and GPs have a gatekeeping function so patients need a referral from primary care to access specialist care. Norway has a universal and publicly financed healthcare system, which is free for all children up to age 16 (18 for mental health services). For adults outpatient health services are subsidized and inpatient care is free. Nearly all specialist care and GP costs are included in the Norwegian Patient Register (NPR) and The Norwegian Control and Distribution of Health Reimbursement Database (KUHR). The NPR also contains information about utilization of private institutions and specialists that are contracted to the public healthcare system [33]. Some healthcare costs are not included in our data, and these include for example: physiotherapy, nursing home costs, and dental care.

We used a de-identified key to link the data from HUNT with data from the NPR and the KUHR database. Norwegian hospitals are partly reimbursed via activity-based financing, and these reimbursements are based on the Diagnosis Related Group (DRG)-system. Our specialist care data included data on somatic, psychiatric, and substance abuse-related inpatient and outpatient contacts that occurred from 2009 to 2016. For episodes of care for which DRG-weights were available, we multiplied the DRG-weight pertaining to each particular episode by the average price of a DRG [34–41] (i.e. average patient cost) for the year during which the DRG was registered. Where DRG-weights were not available (such as for psychiatric contacts) we used data on the average cost of similar contacts for that particular year (details are available in the Additional information). We then summed the costs for each year per patient for the years that the patient was alive and living in Norway, and adjusted the yearly costs to 2016 price levels [42, 43]. Finally, we calculated the average yearly specialist cost per patient during the study period.

The KUHR database contains all electronic patient claims made by general practitioners, and includes information about reimbursed amounts, and patient co-payments for all consultations. To compute GP costs we summed reimbursed amounts and co-payments for each patient for each year between 2009 and 2016 that the patient was alive and living in Norway. We then adjusted the costs to 2016 price levels, and calculated the average yearly cost per patient. All costs were converted from Norwegian Kroner (NOK) to 2016 Euros (1.00 € = NOK 9.29) [44].

#### Data on education, registration status, and relatedness

Next, we linked our dataset with data on educational level from the NUDB database. Age, sex, marital status, urbanity, smoking status, and BMI was measured during the HUNT studies, and were therefore registered at approximately the same time as BMI was measured. For consistency we therefore used education in 1996 for those with a BMI registered in HUNT 2 and educational level registered in 2007 for those whose BMI was reported in HUNT3. The dataset was then linked with data from the Norwegian Population Register on registration status, and individuals were included only during the years that they were alive and living in Norway. Finally, our data were combined with data on relatedness, which allowed us to identify individuals with the same parents.

### Regression models

We investigated the effect of BMI on average yearly GP costs, specialist costs, and total costs using a naïve OLS model, and using a 2SLS IV-regression using genetic variants as instruments. The *ivregress* function in STATA was used to conduct the 2SLS analyses. In the first stage of these analyses, we regressed BMI on each of the instruments (Z) and the control variables (Sex, birthyear, and study (a dummy variable indicating whether participants’ BMI was measured in HUNT 2 or HUNT 3.) (Eq. 1)

1 $${BMI}_i={\pi}_0+{\pi}_1{Z}_i+{\pi}_2{Sex}_i+{\pi}_3{birthyear}_i+{\pi}_4{study}_i+{v}_i,$$

Then, in the second-stage (Eq. 2), we used the predicted values from the first-stage to estimate healthcare costs in the second stage, using the same control variables. The standard errors were corrected for in the two-step procedure.

2 $${HCC}_i={\beta}_0+{\beta}_1{\hat{BMI}}_i+{\beta}_2{Sex}_i+{\beta}_3{birthyear}_i+{\beta}_4{study}_i+{u}_i,$$

The analyses were performed with and without sex-stratification. Sex-stratification was done because it is known that BMI affects the health of males and females differently. However, stratification requires more power, and interventions and treatments are often independent of sex.

Since genetic variants are randomly allocated at conception conditional on parental genes, genetic instruments should not be confounded by other variables. However, the relationship between genetic factors and phenotype expression is not fully understood [45], and if adjusting for potential confounders alters the findings then this could indicate that there is a problem with the instrument, and could warrant further investigation. Also, adjusting for confounders might reduce the residual variability of the dependent variable. We explored adjusting for: i) study period (HUNT 2 or HUNT 3), data years (years of data each participant was residing in Norway) (categorical: 1–8 years), birth year (categorical: years 1906–1989), and sex (categorical), and ii) study period, data years, birth year, sex, educational level (categorical), marital status (categorical), smoking status (categorical), and urbanity (categorical). These particular variables were selected since these are typically included as potential confounders in analyses assessing the effect of BMI on healthcare costs [14]. In the MR analyses, we also explored the effect of adjusting for the first 10 principal components, to adjust for potential population stratification. The statistical power to detect an effect of BMI on GP-, and specialist- cost given our sample of 60,786 individuals was estimated using the mRnd power calculator [46]. Details of the power analyses are available in the additional information and the results are depicted in Fig. S1.

### The genetic instruments

Locke et al., 2015 [29] present 97 genetic variants that have been found to be associated with BMI in genome wide association studies (GWAS), and report the strength of the association between each of these variants and BMI. Only one of these variants (rs12016871) were unavailable in our dataset, and for this variant we followed Brandkvist et al., 2019 [47], and used variant rs4771122 as a proxy. IV-analyses were conducted with three instruments: Instrument 1: An unweighted genetic risk score (GRS) based on the sum of the number of BMI-increasing alleles, out of the 97 BMI-increasing alleles, for each participant. Instrument 2: A weighted GRS which was computed by multiplying the number of BMI-increasing alleles by the respective beta-coefficients from the study by Locke et al., 2015 [29], and then summing the product for each participant. For the sex-stratified analyses we developed sex-specific weighted GRSs using the sex-specific beta-coefficients reported by Locke et al., 2015 [29]. Instrument 3: Including the two genetic variants with the strongest association with BMI (FTO (rs1558902) and MC4R (rs6567160) as dummy variables.

### Instrument validity

Three conditions must be satisfied for an instrument to be regarded as valid.

1. The instrument must be highly correlated with the variables being instrumented, conditional on the other variables in the model (the relevance assumption). In the GWAS that we used to select genetic variants for our instrument, each of the genetic variants were found to be associated with BMI [29], and this has been confirmed in a more recent GWAS [48]. However, the strength of the correlation between each genetic variant and BMI varied between the genetic variants. An instrument can be considered weak if the first-stage F-statistic in the IV-regressions is smaller than 10 (F < 10) [49]. A weak instrument will bias the 2SLS estimates towards the OLS estimate [50]. Combining genetic variants into a GRS, as we have done in this study, leads to a higher first-stage F-statistic, and reduced bias [51].
2. There should not be any omitted variables (measured or unmeasured) on the pathway between the instrument and healthcare costs (the independence assumption). The allocation of genes at conception can be regarded as random, and therefore we assume that the independence assumption largely holds. However, there are some potential problems, for example: i) mating can be non-random since individuals with similar phenotypes are more likely to mate (assortative mating), ii) genes are conditional on parents genes (dynastic effects), iii) one or more non-confounding variables can modify the effect of the genetic variants on healthcare costs (effect modification), iv) some allele variants are more likely to be inherited together than one would expect from chance (linkage disequilibrium) and if these overrepresented alleles lead to increased healthcare costs through pathways that are unrelated to BMI, then the study is likely to be biased [51]. And v) there are differences in the allele-frequencies of particular population sub-groups due to differential ancestry (population stratification) [52]. The independence assumption is not fully testable, but some methods, that will be described later, have been developed to assess some of the factors that can lead to violations of the independence assumption.
3. Each genetic variant should only affect healthcare costs via BMI (the exclusion restriction). This condition cannot be verified and will be violated if genetic variants that have been shown to be associated with BMI are simultaneously associated with other phenotypic traits that are unrelated to BMI (horizontal pleiotropic effects), but related to healthcare costs. Since the biological understanding of each of the genetic variants used in our study is limited, the extent of horizontal pleiotropy is unknown. Other possible violations of the exclusion restriction include, but are not limited to: linkage disequilibrium, population stratification, and effect modification. In the next section, we will describe analyses that we have done to investigate the potential violations of the IV-assumptions.

### Sensitivity analyses

To investigate the possibility of bias in our estimates we conducted a series of sensitivity analyses, including two-sample methods, non-linear analyses, sex-, healthcare provider-, and age-specific analyses, within-family analyses, and outlier removal.

#### Two sample methods

The two-sample MR methods involved combining summary data on the genetic variant-BMI association from Locke et al., 2015 [29] with data on the association between the genetic variants and GP-, specialist-, and total- costs, estimated from our data (Additional information, Table S1). The overlap between our sample and the data used by Locke et al., 2015 [29] was minimal, and both studies provide results from samples with similar ancestries (European descent), and adjust for age and sex.. When the samples used in two-sample analyses are independent, the two-sample estimates will be biased towards zero, rather than the observational estimate [53].

We began by testing for heterogeneity of the genetic instruments using Cochran’s Q test. If there is more heterogeneity than one would expect from chance, then this might indicate violation of the IV-assumptions, for instance due to horizontal pleiotropy [54].

We further evaluate heterogeneity by applying the following two-sample methods: Inverse variance weighted estimation (IVW), MR-Egger regression (including MR-Egger with a SIMEX (Simulation Extrapolation correction), weighted median estimation, and weighted mode-based estimation. These methods primarily examine violations of the exclusion restriction, particularly horizontal pleiotropy. The methods rely on different assumptions that can only be partially tested. Thus, comparing findings from the different methods is advantageous as this can reveal various potential threats to the IV-assumptions.

The Inverse variance weighted (IVW) method is a weighted linear regression of the summary SNP-healthcare cost association on the summary SNP-BMI associations, with the intercept constrained to zero [55, 56]. The IVW estimate is similar to the 2SLS estimate, and will be a poor estimate if there is bias due to pleiotropy. The IVW method requires that any directional (non-zero) pleiotropic effects on the outcome are independent of the SNP-BMI associations, and the NOME assumption, that there is no measurement error in the SNPs exposure association, to hold.

The MR-Egger method is similar to the IVW method, but the intercept is not constrained to pass through zero [54]. MR-Egger requires that the InSIDE (instrument strength is independent of direct effects) and NOME assumptions hold [55]. Bias due to measurement error was estimated using the regression dilution statistic (${I}_{GX}^2$). If ${I}_{GX}^2$ was < 90%, we used the SIMEX (Simulation Extrapolation) correction [55]. When this correction is applied the SNP-BMI associations are estimated in repeated simulations before they are combined with the SNP-healthcare cost associations [55]. It is not possible to test the InSIDE assumption, but if it holds the slope estimated from MR-Egger regression can be interpreted as the true estimate under pleiotropy [54], and the intercept can be interpreted as an estimate of the average pleiotropic effect across the instruments [55]. If the InSIDE assumption is violated and there exists directional pleiotropy, then the MR-Egger estimate may be biased. The MR-Egger estimates are less precise and have low power [57].

The weighted median approach uses the median of the inverse-variance weighted ratio estimates [57, 58]. This method is more robust to outliers than IVW and MR-Egger, and provides a consistent estimate if at least 50% of the weight comes from genetic variants that are valid instruments [57].

The weighted mode approach uses the mode of the inverse-variance weighted ratio estimates [58]. This approach is more powerful than the MR-Egger and less powerful than the IVW and weighted-median approaches, and will give a consistent estimate if the largest weights originate from valid genetic variants [59]. This method requires that the zero modal pleiotropy assumption (ZEMPA) (i.e. that the most common effect is a consistent estimate of the true causal effect) holds [59]. This assumption was assessed by constructing density plots and inspecting these fore multiple peaks [59].

To ease interpretation of our findings, we followed Budu-Aggrey et al., 2019 [60] A, and Dixon et al., 2020 [27], and transformed the two-sample estimates to natural BMI-units by dividing the estimates by the median standard deviation (4.6) of BMI reported by Locke et al., 2015 [29]. As a result, the estimates from the two-sample methods can be interpreted as the marginal effect of a one-unit increase in BMI on healthcare costs.

#### Some invalid some valid instrumental variables estimator

We used the sisVIVE (Some Invalid Some Valid Instrumental Variables Estimator), which uses LASSO to identify potentially invalid instruments, and to estimate a causal effect of the exposure on the outcome in the presence of invalid instruments [61]. First, we estimated the most appropriate lambda using ten-fold cross validation, then we use this lambda in estimating the effect estimate and potential invalid instruments. This was done using the sisVIVE package in R [61].

#### Non-linear analyses

The associations between BMI and healthcare outcomes may be nonlinear [25, 62, 63]. Genetic variants explain a relatively small proportion of variance in BMI, and therefore non-linear effects might be challenging to detect. We used a method proposed by Staley and Burgess, 2017 [64] to assess non-linearity in studies using genetic variants as instruments. The method includes two tests for nonlinearity: a quadratic and a fractional polynomial test. The sample was divided into 10 strata using residual BMI, and then linear IV-regression estimates were calculated for each stratum by dividing the association between the GRS and each healthcare cost outcome by the association between the GRS and BMI. Next, a meta-regression was performed, where the estimated values for each stratum are regressed against the mean of BMI in each stratum using a flexible semiparametric framework.

#### Stratified analyses

We performed analyses stratified by sex, and specialist healthcare provider (somatic hospital care, psychiatric hospital care, providers of somatic and psychiatric care that were contracted to specialist care, and contacts related to interdisciplinary specialized drug treatment).

#### Within family analyses

These analyses can provide information about the effect of possible violations of the IV-assumptions due to assortative mating, dynastic effects and population stratification [65]. There are several proposed methodological variations of within-family analyses [65]. We conducted a 2SLS regression with family-fixed effects. These analyses require more power than population-based methods.

#### Outlier removal

We identified outlying genetic variants from forest plots of the effect of each of the genetic variants on GP- and specialist costs. Next, we used the the PhenoScanner database [66], to check if these genetic variants had been found to be associated with non-BMI related phenotypes. Then we explored the effect of removing the outermost outliers from the analysis. If removal of an outlier substantially alters the estimators, and the removed variant is an invalid instrument, then including this variant in our instrument may have biased the results.

### Ethical approval

The study was approved by the Regional Committee for Ethics in medical research (2016/537/REK midt).

### Software

STATA 15 was used for the regression analyses and R version 3.4.1 for data processing. The MR robust package [67] was used for two-sample MR analyses.

## Results

Our dataset contained information about BMI, genetic variants related to BMI, sociodemographic variables (Table 1), GP-, specialist- and total costs (Table 1) for 60,786 individuals.

**Table 1:** Descriptive information by sex

Variable	Category	Males & females	Sex	
			Male	Female
		N (%)	N (%)	N (%)
Total		60,786 (100.0)	28,136 (100.0)	32,650 (100.0)
Age	18–24	4090 (6.9)	1843 (6.7)	2247 (6.6)
	25–44	18,506 (30.4)	8587 (30.4)	9919 (30.5)
	45–66	26,389 (42.4)	12,553 (43.4)	13,836 (44.6)
	67–79	9554 (16.2)	4256 (15.7)	5298 (15.1)
	80+	2247 (4.1)	897 (3.7)	1350 (3.2)
	Missing	0 (0.0)	0 (0.0)	0 (0.0)
Marital Status	Married/ Registered partner	34,225 (54.5)	16,432 (56.3)	17,793 (58.4)
	Unmarried	15,938 (23.3)	8321 (26.2)	7617 (29.6)
	Divorced/separated	5786 (10.1)	2475 (9.5)	3311 (8.8)
	Widow/Widower	4749 (11.9)	866 (7.8)	3883 (3.1)
	Missing	88 (0.1)	42 (0.1)	46 (0.1)
Education	Primary school	14,362 (25.7)	5978 (23.6)	8384 (21.2)
	Secondary school	31,114 (46.2)	16,027 (51.2)	15,087 (57)
	Higher Education. short	12,584 (25.0)	4420 (20.7)	8164 (15.7)
	Higher Education. long	2571 (2.8)	1651 (4.2)	920 (5.9)
	Missing	155 (0.3)	60 (0.3)	95 (0.2)
Urbanity	Urban	39,362 (64.8)	18,197 (64.8)	21,165 (64.7)
	Rural	21,124 (34.6)	9825 (34.8)	11,299 (34.9)
	Missing	300 (0.6)	114 (0.5)	186 (0.4)
Smoking status	Smoker	13,141 (23.1)	5589 (21.6)	7552 (19.9)
	Former smoker	18,347 (27.2)	9474 (30.2)	8873 (33.7)
	Never smoker	25,184 (43.4)	11,030 (41.4)	14,154 (39.2)
	Missing	4114 (6.3)	2043 (6.8)	2071 (7.3)
BMI category^a^	Underweight	373 (0.9)	82 (0.6)	291 (0.3)
	Normal weight	20,451 (38.8)	7777 (33.6)	12674 (27.6)
	Overweight	26,612 (37.3)	14,431 (43.8)	12,181 (51.3)
	Class I Obesity	10,243 (16.5)	4869 (16.9)	5374 (17.3)
	Class II Obesity	3107 (6.5)	977 (5.1)	2130 (3.5)
	Missing	0 (0.0)	0 (0.0)	0 (0.0)

^a^The BMI-categories were defined as follows: Underweight = BMI < 18.5, normal weight = BMI: 18.5–24.9, overweight = BMI: 25.0–29.9, class I obesity = BMI: 30.0–34.9, and class II obesity = BMI ≥35.0

The mean age of participants at BMI measurement was 51.1 years (S.D = 16.3) for males and 51.4 years (S.D = 16.9) for females. The average BMI of the participants was 27.2 kg/m^2^ (S.D = 3.8) for males, and 26.9 kg/m^2^ (S.D = 4.9) for females. During the eight-year study period, 98.7% (*n* = 27,775) of males, and 99.2% (*n* = 32,397) of females had incurred costs for GP services, and 68.2% (*n* = 19,180) of males, and 69.2% (*n* = 22,588) of females had incurred costs for specialist services. The average crude GP- and specialist costs generally increased with BMI, but costs were also high for males and females with underweight (Table 2). The average yearly GP cost was € 161.8 (S.D = 177.7, median = 108.1) for males, and € 196.5 (S.D = 182.7, median = 149.8) for females. The average yearly specialist cost was, € 2233.9 (S.D = 6698.0, median = 226.4) for males, and € 2066.1 (S.D = 5702.0, median = 370.9) for females (Table 2).

**Table 2:** Mean (standard deviation (S.D)) crude sex- and BMI-specific yearly healthcare costs

Type of healthcare	BMI-category^a^	Males & females	Sex	
			Male	Female
		Mean (S.D) (€)	Mean (S.D) (€)	Mean (S.D) (€)
GP costs	Underweight	201.7 (216.8)	122.2 (121.2)	224.1 (232.1)
	Normal weight	154.4 (163.9)	134.7 (165.8)	166.4 (161.5)
	Overweight	175.3 (173.1)	157.3 (166.8)	196.6 (177.9)
	Class I Obesity	216.1 (199.3)	198.1 (195.3)	232.4 (201.5)
	Class II Obesity	276.5 (233.3)	267.9 (253.9)	280.4 (223.2)
Specialist Costs	Underweight	3454.3 (10,268.7)	2496.0 (6469.4)	3724.2 (11,101.2)
	Normal weight	1804.9 (5616.0)	1949.7 (5982.9)	1716.1 (5376.6)
	Overweight	2130.9 (6193.9)	2225.4 (7046.7)	2019.0 (4996.4)
	Class I Obesity	2481.4 (6536.6)	2387.8 (5839.9)	2566.2 (7108.5)
	Class II Obesity	3214.1 (7479.6)	3831.7 (9849.7)	2930.8 (6073.2)
Total costs	Underweight	3656.0 (10,343.2)	2618.3 (6517.7)	3948.4 (11,179.3)
	Normal weight	1959.3 (5667.6)	2084.4 (6040.3)	1882.5 (5425.1)
	Overweight	2306.2 (6247.1)	2382.7 (7096.4)	2215.5 (5058.6)
	Class I Obesity	2697.5 (6602.6)	2585.9 (5915.0)	2798.6 (7167.9)
	Class II Obesity	3490 (7556.8)	4099.6 (9934.7)	3211.2 (6149.5)

^a^The BMI-categories were defined as follows: Underweight = BMI < 18.5, normal weight = BMI: 18.5–24.9, overweight = BMI: 25.0–29.9, class I obesity = BMI: 30.0–34.9, and class II obesity = BMI ≥35.0

### Naïve OLS regression

Increased BMI was significantly associated with higher costs for males and females, and with and without adjusting for potential confounding variables (Table 3).

**Table 3:** OLS estimates of the effect of BMI on GP-, specialist- and total costs

Type of healthcare costs	Adjustment	Males & females	Males	Females
		Beta (SE) (€)	Beta (SE) (€)	Beta (SE) (€)
General practitioner costs	I^a^	5.8 (0.16)***	7.0 (0.26)***	5.5 (0.20)***
	II^b^	5.8 (0.16)***	6.9 (0.26)***	5.5 (0.20)***
Specialist costs	I	44.6 (5.40)***	50.8 (9.91)***	45.2 (6.24)***
	II	52.0 (5.48)***	57.9 (10.04)***	50.8 (6.34)***
Total costs	I	45.7 (5.40)***	52.1 (9.91)***	46.2 (6.24)***
	II	53.0 (5.48)***	59.1 (10.05)***	51.8 (6.34)***

*** (*p*-value < 0.001)

^a^ Estimates were adjusted for study period, years of data available, birth year, and sex

^b^ Estimates were adjusted for study period, years of data, birth year, sex, educational level, smoking status, marital status, and urbanity

### 2SLS IV regressions

The F-statistic was high, and well above 10, for the three instruments in all the analyses, indicating that our instruments were strong (Table 4). We found a positive association between BMI and GP costs when using the unweighted (*p*-value< 0.001) and weighted GRSs (*p*-value< 0.001), and found no evidence of an association (*p*-value = 0.398) when using the FTO/MC4R instrument. The finding was consistent when potential covariates were added to the model (Additional information, Table S2), and when the first 10 principal components were added (Additional information, Table S3).

**Table 4:** 2SLS estimates^a^ of the effect of BMI on GP-, specialist-, and total costs

Type of healthcare cost	Instrument	2SLS First-stage Beta instrument (SE)	2SLS Second-stage Beta BMI (SE) €	
				F-stat.
General practitioner costs	Unweighted GRS	0.1 (0.003)***	6.6 (1.177)***	1123.0
	Weighted GRS	4.0 (0.105)***	5.6 (1.026)***	1484.4
	FTO & MC4R	0.4 (0.025)*** & 0.3 (0.028)***	1.8 (2.116)	173.2
Specialist costs	Unweighted GRS	0.1 (0.003)***	2.3 (40.035)	1123.0
	Weighted GRS	4.0 (0.105)***	14.9 (34.915)	1484.4
	FTO & MC4R	0.4 (0.025)*** & 0.3 (0.028)***	−0.3 (71.640)	173.2
Total costs	Unweighted GRS	0.1 (0.003)***	3.3 (40.053)	1123.0
	Weighted GRS	4.0 (0.105)***	15.8 (34.932)	1484.4
	FTO & MC4R	0.4 (0.025)*** & 0.3 (0.028)***	−0.4 (71.675)	173.2

*** (*p*-value < 0.001)

^a^ The estimates were adjusted for study period (HUNT 2 or HUNT 3), years of data participants were alive and living in the country during the cost estimation period (2009–2016), sex, and birth year

Compared with the OLS estimates (Table 3) adjusted for study period, years of data, sex, and birth year, the 2SLS effect estimates were 14% larger when using the unweighted GRS, 3% smaller when using the weighted GRS, and 69% smaller when using the FTO and MC4R instrument.

We did not find evidence of an association between BMI and specialist costs or between BMI and total costs, when using any of the three combinations of genetic variants as instruments. However, this could be due to a lack of power. The 2SLS coefficients (Table 4) were smaller, and had larger standard errors, compared with the OLS coefficients (Table 3). The same was found when adjusting for more potential covariates (Additional information, Table S2) and when adjusting for the first 10 principal components (Additional information, Table S3). The estimated coefficients when adjusting for either potential covariates, or the first 10 principal components were similar to the coefficients estimates in the main 2SLS analysis (adjusted for study period, years of data available, sex, and birth year).

### Sensitivity analyses

#### Two-sample Mendelian randomization

The Cochran’s Q tests indicated that there was heterogeneity when assessing the association between BMI and GP costs (Q = 131.5, *p*-value = 0.008), specialist costs (Q = 129.1, *p*-value = 0.012), and total costs (Q = 128.5, *p*-value = 0.013). Heterogeneity might suggest violations of the IV assumptions, for instance through horizontal pleiotropy.

The results of the two-sample methods are presented and together with the OLS and one-sample estimates. The regression dilution statistic (${I}_{GX}^2$) was 89.8, indicating that the MR-Egger estimate had a 10.2% relative bias towards the null. Since the bias was greater than 10%, we also conducted an MR-Egger with SIMEX to adjust for attenuation bias.

**Fig. 2:**
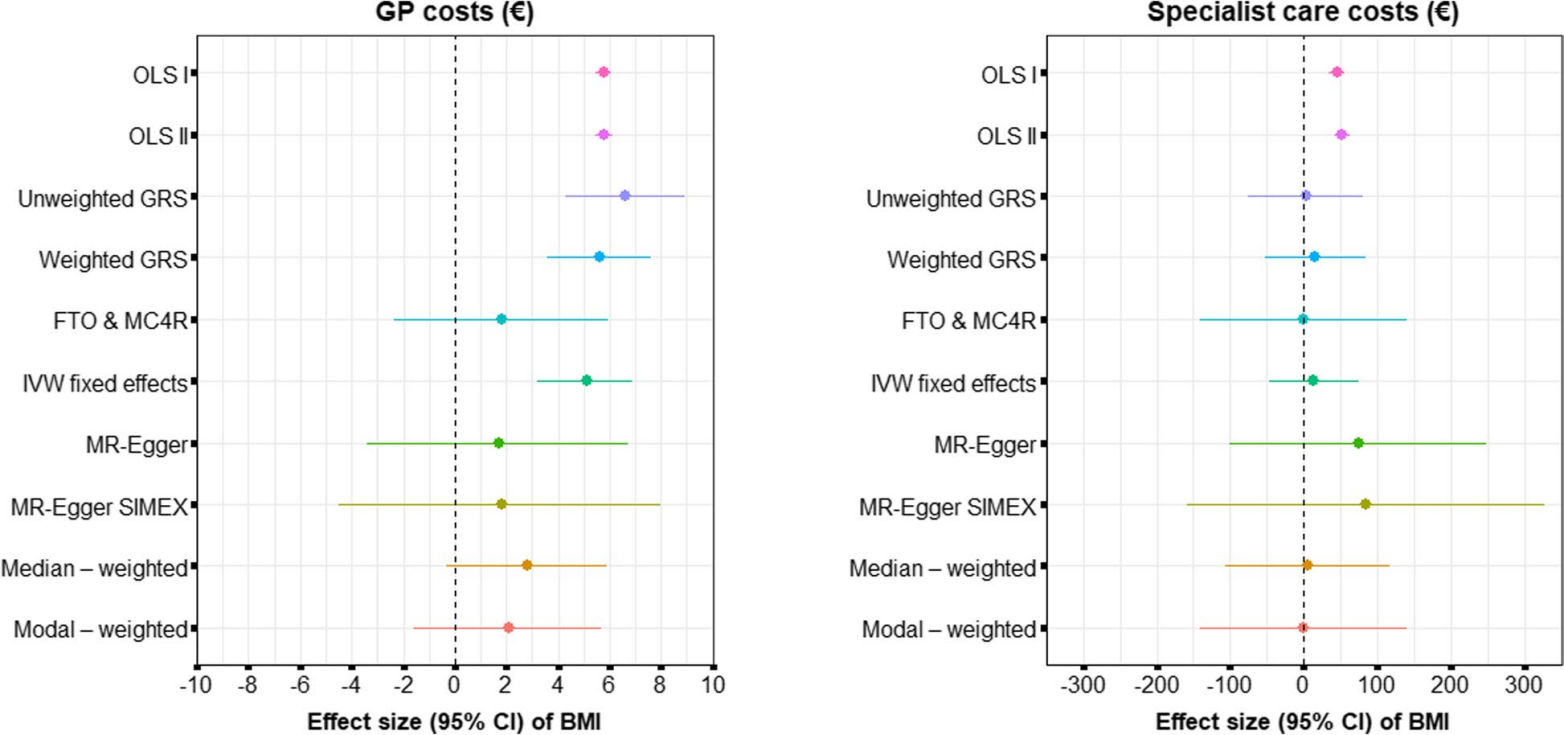
Estimates and 95% confidence intervals (CIs) of the effect of BMI on GP (left) and specialist (right) costs using OLS, one-sample, and two-sample methods

For GP costs, there seemed to be some bias due to horizontal pleiotropy. The MR-Egger, median- and mode- based estimates also suggested that the true (pleiotropy-adjusted) estimate was below the IVW estimate (Fig. 2, Additional information, Fig. S3A). Evidence of horizontal pleiotropy was also found when plotting the effect-sizes for each genetic variant against the strength of the association between each genetic variant and BMI in a funnel plot (Additional information, Fig. S2A).

We had no way of ascertaining whether the assumptions required for the MR-Egger estimate to hold were violated, and we have no reason to believe that the median-based estimate was unreliable. The modal-based estimate, however, should be interpreted with some caution, as we found some indication of modal pleiotropy (Additional information, Fig. S6A). The *p*-values for all the two-sample estimates, except the IVW estimate, were weakly or not significant (Fig. 2), but this could be due to a lack of power since these methods require more power to detect an effect than the one-sample methods.

For specialist costs, there were also some indications of horizontal pleiotropy (Fig. 2, Additional information (Fig. S3B), but the different two-sample methods indicated pleiotropy in different directions. The MR-Egger estimates suggested that the true association between BMI and specialist costs was higher than the IVW estimate. The median- and modal-based estimates, however, indicated that the true effect was smaller than the IVW estimate. The modal plot exhibited some evidence of modal pleiotropy (Fig. S6B). Visual inspection of the funnel plot (Additional information, Fig. S2B) indicated slight asymmetry indicating that the true value could be smaller than the IVW estimate. None of the two-sample estimates for specialist costs were statistically significant, but we had limited power to detect an effect. Since total costs were largely driven by specialist costs, the results for total costs were similar to those reported for specialist costs.

#### Some invalid some valid instrumental variables estimator

The sisVIVE did not identify any invalid instruments in any of the analyses. For GP costs (Additional Information, Table S4), the coefficients were similar to the results from the 2SLS analyses, and also corresponded well with the 2SLS results in that the estimated coefficients were slightly lower for females than for males. Although sisVIVE was not able to detect any invalid instruments, the results indicate that potentially invalid instruments may affect the healthcare costs of males and females differently. As the number of invalid instruments increased, the estimated coefficient became higher for males and lower for females. For specialist costs the results for males and females together were larger than the 2SLS estimates, and similar to the OLS estimates.

#### Non-linear analyses

There was some evidence of nonlinearity in the association between BMI and GP-costs (Additional information, Fig. S7). The *p*-values from the quadratic and fractional polynomial tests were both < 0.05. The corresponding tests for the association between BMI and specialist costs did not suggest non-linearity.

#### Sex, healthcare provider, and age specific analyses

In the 2SLS analyses, we found some indications that healthcare costs differed between males and females (Additional information, Table S5). For both males and females, we found an association between BMI and GP costs when using the unweighted and weighted GRSs (*p*-value< 0.0001 for males and females), but not when using the FTO & MC4R instrument (*p*-value = 0.386 for males, *p*-value = 0.075 for females). For both males and females, the second-stage coefficients when using the GRSs as instruments were similar to the OLS estimates, and the coefficients were smaller when using the FTO & MC4R instrument.

For both males and females, we found no evidence of an association between BMI and specialist costs when using the three different instruments. Compared with the OLS estimates, the 2SLS coefficients were larger for males, and smaller (and negative) for females. However, we likely had insufficient power to detect effects of this size. Again, the results for total costs were similar to the results for specialist costs.

Stratifying specialist costs based on the healthcare provider indicated that the effects observed for specialist costs were mainly driven by costs incurred by patients that had received somatic care in hospitals (Additional information, Table S6). As with specialist costs overall, we found no significant association between BMI and healthcare costs, for any of the different healthcare providers. For costs incurred in somatic hospitals and for contacts with specialists on contract with specialist care providers, we found a negative effect (not significant) of BMI on costs for females, and a positive effect (significant only for somatic hospital costs when using the weighted GRS instrument) of BMI on costs for males.

#### Outlier removal

For GP costs, we found three (rs4740619, rs13191362, and rs4787491) right-lying outliers (Fig. S4). One of these (rs4787491) had been found to be associated with several potentially confounding phenotypes (such as: age at menarche, time spent driving, employment status, and alcohol intake frequency). Removing one or all of the outliers from the analyses on GP costs (Additional information, Table S7) reduced the effect estimate by up to 14% when using the unweighted GRS, and by up to 11% when using the weighted GRS.

For specialist costs, we identified one (rs7715256) right-lying outlier (Fig. S5). This variant had been found to be associated with age at menarche, and basal metabolic rate, in addition to BMI-related phenotypes. Removing this outlier from specialist costs resulted in lower effect estimates (Additional Information, Table S8), but the effects remained non-significant.

#### Within-family analyses

For GP costs, the results of the within-family analyses were similar to results from the full population analyses (€ 3.9 smaller when using the unweighted and weighted GRSs, and € 1.0 smaller when using the FTO & MC4R instrument) (Additional information, Table S9). For specialist costs the within-family estimates were considerably smaller and negative when using all the instruments (€ 67.9 smaller when using the unweighted GRS, € 98.6 smaller when using the weighted GRS, and € 155.6 smaller when using the FTO and MC4R instrument), compared with the estimates from the full sample. None of the effect estimates were significant. However, since the sample was reduced to 29,199 observations, and 11,723 groups, we might have had insufficient power.

## Discussion

Compared with the naïve OLS estimates we found small and precise estimates of the effect of BMI on GP costs. Our results indicate that a one-unit increase in BMI increases GP costs by less than € 5.6 per year. Assuming linearity and that the difference in BMI between normal weight and obesity is 10.75 kg/m^2^ these results suggest that obesity leads to a maximum of € 60.2 higher GP-cost per year. For specialist costs, the main analyses suggested that the effect of BMI on specialist costs were smaller and less precise than the OLS estimate.

Previous studies assessing the effect of BMI on healthcare costs or utilization have found substantially higher costs and/or utilization when using the BMI of a relative [22–26] or when using genetic variants [27, 28] as instruments in IV-analyses, compared with the costs found using traditional methods. There are several potential explanations for why our results do not follow the same pattern. The studies that have used biological relatives as instruments are not able to rule out environmental effects to the same extent as studies using individuals’ genetic variants as instruments. However, the use of biological relatives as instruments is also more powerful, as it may explain more of the variance in BMI compared with the use of genetic variants [68]. Nevertheless, this approach is limited because it omits individuals without children.

The two existing studies that have used genetic variants as instruments to assess the effect of BMI on healthcare costs both concluded that increased BMI leads to higher healthcare costs. One possible reason why our results do not follow the same trend is that there are differences between the data sources used. Compared with the first study, which was based on UK Biobank data [27], our sample had a larger response rate (54.1% in our data vs. 5.45% in the UK-based study), and included a broader age span (ages 20 and above in our data vs. 99.5% of the sample being between 40 and 69 years of age in the UK-based study). The UK Biobank sample (*n* = 307,048) was larger than our sample (*n* = 60,728). The second study by [28], conducted in South West Germany, had a small sample size for the MR context (*n* = 2796), and the data on healthcare utilization used to estimate costs were self-reported in interviews. Self-reported healthcare utilization could lead to bias, especially if the number of encounters are high [20]. Although the results of our study are not directly comparable with the studies by Kurz and Laxy, 2020 [28] and Dixon et al., 2020 [27], there were some noteworthy discrepancies between our findings. In our study and in the study by Dixon et al., 2020 [27] the findings suggested that BMI had a smaller effect on hospital costs for females than for males. While the findings from the study by Kurz and Laxy, 2020 [28] suggests that BMI may increase healthcare costs more among females. In our study, and in the study by Dixon et al., 2020 [27], the weighted median and modal estimates were smaller than the IVW estimates, while the weighted median estimate was higher than the IVW estimate in the study by Kurz and Laxy, 2020 [28] . Lastly, the MR-Egger estimate was smaller than the IVW estimate in the study by Dixon et al., 2020 [27] and in the study by Kurz and Laxy, 2020 [28], while in our study the MR Egger estimate was higher than the IVW estimate. These discrepancies are potentially relevant for evaluating the reliability of MR-based estimates.

There are also some important limitations of studies using genetic variants as instruments. First, our understanding of BMI is limited, and BMI is trait heterogeneous (i.e. the genetic pathways leading to high BMI are heterogeneous). For instance, some people may have genetic variants that lead to an increased probability of poor diet, others may be predisposed to low metabolism or overeating. Ideally, we should seek to identify genetic variants that are associated with different underlying causes, and assess the individual impact of each of these on BMI and healthcare costs. Second, our power calculations show that we may have had insufficient power to detect small effect sizes, and this may explain why we did not find a significant effect of BMI on specialist costs (Fig. S1). This is an important problem, but at the same time, if obesity is associated with small costs, then the consequences of a type II error are smaller. Third, because our costs were calculated based on the average costs for a particular type of contact, we have not accounted for differences in costs between BMI-categories for the same type of contact. Fourth, although we have done our best to test the validity IV-assumptions, we need an improved understanding of how each of the genetic variants work to fully understand the limitations of their use [69]. Fifth, it is possible that the health consequences of acquiring obesity due to a genetic predisposition, differ from the health consequences of obesity acquired through environmental factors (e.g. social transmission). Lastly, since obesity is associated with early mortality [70], we may have captured a larger proportion of lifetime healthcare costs for persons with obesity, compared with those without. In addition to these limitations, our study lacked a sufficient sample size to conduct a wider range of sensitivity analyses.

If our results are correct then there is a causal effect of increased BMI on GP costs. The effect of BMI on specialist costs is more uncertain, and may be smaller than has been estimated in previous studies. This result is not in line with the findings of the two previous studies that have used the MR methodology to look at specialist costs, and could be due to a lack of power. However, since the two past studies also have important weaknesses future studies should consider applying the Mendelian randomization methodology to investigate whether similar findings are detected when using other data sources. We also found that the BMI-related genetic variants that resulted in higher GP costs (Additional information, Fig. S4) were not the same as the ones driving specialist costs (Additional information, Fig. S5). Moreover, we found that different genetic variants were drivers of healthcare costs for males and females, and for different types of specialist care. Lastly, when using the GRSs the results were sometimes different compared with when we used the variants with the strongest association with BMI (FTO and MC4R). These findings might suggest that there are differences in the supply and/or demand of healthcare services for individuals with different genetic variants or groups of genetic variants. This could be related to the many different potential pathways leading to obesity, and increased knowledge on this topic could be relevant for evaluations of prevention and treatment strategies.

The differing results found for males and females may also be explained by biological differences. National data on the number of sex-specific healthcare contacts made suggests that contact frequency is often higher for females than for males for diagnoses that are typically related to BMI (such as heart disease, diabetes, and circulatory diseases) (Additional information, Fig. S8).

Since our study and all similar studies have different sample-related limitations, it will be important to conduct more studies, ideally with nationally representative data with a large sample size, and a broad age-span on participants. Future studies should also include as many newly discovered BMI-related genetic variants as possible. Since the initiation of the current study two new GWAS have revealed additional BMI-related genetic variants [48, 71]. Finally, it could also be interesting to further investigate differences between the OLS and 2SLS estimates to try to uncover potential sources of simultaneity bias, for instance by comparing effect estimates for specific types of healthcare contact.

## Conclusion

The effect of BMI on healthcare cost attenuated in the IV models, compared with the naïve OLS models. Previous studies may have overestimated the effects of obesity on GP and specialist costs.

## Supplementary Information

**Additional file 1.**

## Data Availability

The data that support the findings of this study are available from third parties. Restrictions apply to the availability of these data, which were used under license for this study. Please contact the corresponding author for more information about data accessibility.

## Abbreviations

BMI: Body Mass Index
HUNT: Nord-Trøndelag Health Studies
GP: General Practitioner
IV: Instrumental Variable
OLS: Ordinary Least Squares
MR: Mendelian randomization
NPR: Norwegian Patient Registry
KUHR: Norwegian Control and Payment of Health Reimbursement
DRG: Diagnosis Related Group
NOK: Norwegian Kroner
NUDB: National Education Database
2SLS: Two-Stage Least Squares
GWAS: Genome Wide Association Study
