# Supplementary material for: Body mass index and healthcare costs: using genetic variants from the HUNT study as instrumental variables
Source: BMC Health Serv Res. 2022 Mar 25;22:396. doi: 10.1186/s12913-022-07597-z (PMC8957125; doi:10.1186/s12913-022-07597-z)
Supplement: Supplementary file 1 — Additional file 1. [file 12913_2022_7597_MOESM1_ESM.docx]

**Body mass index and healthcare costs: using genetic variants from the HUNT study as instrumental variables**

**Additional information**

**Overview of tables included in the additional information file**

[**Table S1: The regressed effect of each of the SNPs on primary care and secondary care costs. The values are adjusted for birthyear and sex.** 14](#_Toc89435722)

[**Table S2: Results from the one-sample 2SLS regressions for each type of healthcare cost and using three different instruments. As a sensitivity analysis, the estimates were adjusted for study period, years of data for which participants were alive and living in Norway, birth year, sex, educational level, marital status, smoking status, and urbanity.** 18](#_Toc89435723)

[**Table S3: Beta coefficients and standard errors (SE) for the first- and second-stage results of the one-sample 2SLS regressions**^†^ **for each type of healthcare cost, using three different instruments. The estimates were adjusted for study period (HUNT 2 or HUNT 3), years of data participants were alive and living in the country during the cost estimation period (2009-2016), sex, birth year, and the first 10 genetic principal components.** 19](#_Toc89435724)

[**Table S4: The estimated effect of BMI on GP-costs and specialist costs for both sexes, and for males and females, with 0 or 96 invalid instruments.** 20](#_Toc89435725)

[**Table S5: Sex-specific, first- and second-stage results of the one-sample 2SLS regressions for each type of healthcare cost, using three different instruments. The estimates were adjusted for study period (HUNT 2 or HUNT 3), years of data participants were alive and living in the country during the cost estimation period (2009-2016), and birth year.** 21](#_Toc89435726)

[**Table S6: Sex-specific, first- and second-stage results of the one-sample 2SLS regressions for specialist costs by type of healthcare provider, using three different instruments. The estimates were adjusted for study period (HUNT 2 or HUNT 3), years of data participants were alive and living in the country during the cost estimation period (2009-2016), and birth year.** 22](#_Toc89435727)

[**Table S7: First- and second-stage results of the one-sample 2SLS regressions for GP costs with outliers excluded, using three different instruments. The estimates were adjusted for study period (HUNT 2 or HUNT 3), years of data participants were alive and living in the country during the cost estimation period (2009-2016), sex, and birth year.** 23](#_Toc89435728)

[**Table S8: First- and second-stage results of the one-sample 2SLS regressions for specialist costs with the outlier excluded, using three different instruments. The estimates were adjusted for study period (HUNT 2 or HUNT 3), years of data participants were alive and living in the country during the cost estimation period (2009-2016), sex, and birth year.** 23](#_Toc89435729)

[**Table S9: First- and second-stage results of the one-sample 2SLS regressions for GP- and specialist costs within families (N=29 199), using three different instruments. The estimates were adjusted for study period (HUNT 2 or HUNT 3), years of data participants were alive and living in the country during the cost estimation period (2009-2016), sex, and birth year.** 24](#_Toc89435730)

**Genotyping procedures**

The genotyping was conducted by the K.G. Jebsen Center for Genetic Epidemiology, Department of Public Health and Nursing, Faculty of Medicine and Health Sciences, University of Science and Technology (NTNU), Trondheim, Norway. There is no formal publicly available document describing the quality control and imputation procedures conducted. However, the main procedures conducted are described in a fact sheet provided by the K.G. Jebsen Center.

“**Quality Control**

In total, DNA from 71,860 HUNT samples was genotyped using one of three different Illumin HumanCoreExome arrays (HumanCoreExome12 v1.0, HumanCoreExome12 v1.1 and UM HUNT Biobank v1.0). Samples that failed to reach a 99% call rate, had contamination > 2.5% as estimated with BAF Regress [1], large chromosomal copy number variants, lower call rate of a technical duplicate pair an twins, gonosomal constellations other than XX and XY, or whose inferred sex contradicted the reported gender, were excluded. Samples that passed quality control were analysed in a second round of genotype calling following the Genome Studio quality control protocol described elsewhere [2]. Genomic position, strand orientation and the reference allele of genotyped variants were determined by aligning their probe sequences against the human genome (Genome Reference Consortium Human genome build 37 and revised Cambridge Reference Sequence of the human mitochondrial DNA; http://genome.ucsc.edu) using BLAT [3]. Variants were excluded if (1) their probe sequences could not be perfectly mapped to the reference genome, cluster separation was < 0.3, Gentrain score was < 0.15, showed deviations from Hardy Weinberg equilibrium in unrelated samples of European ancestry with p‐value < 0.0001), their call rate was < 99%, or another assay with higher call rate genotyped the same variant.”

“**Ancestry/Population structures**

What info can you get in your file?

There is no individual level information about ancestry or population structure in the exported file. In the quality control of the data‐set the following has been done: Ancestry of all samples was inferred by projecting all genotyped samples into the space of the principal components of the Human Genome Diversity Project (HGDP) reference panel (938 unrelated individuals; downloaded from http://csg.sph.umich.edu/chaolong/LASER/) [4,5], using PLINK v1.90 [6]. Recent European ancestry was defined as samples that fell into an ellipsoid spanning exclusively European populations of the HGDP panel. The different arrays were harmonized by reducing to a set of overlapping variants and excluding variants that showed frequency differences > 15% between data sets, or that were monomorphic in one and had MAF > 1% in another data set. The resulting genotype data were phased using Eagle2 v2.3 [7].”

“**Imputation**

What info can you get in your file?

There is no individual SNP‐level information about imputation quality in the exported file. In the imputation and quality control of the data‐set the following has been done: Imputation was performed on the 69,716 samples of recent European ancestry using Minimac3 (v2.0.1,

http://genome.sph.umich.edu/wiki/Minimac3) [8] with default settings (2.5 Mb reference based chunking with 500kb windows) and a customized Haplotype Reference consortium release 1.1 (HRC v1.1) for autosomal variants and HRC v1.1 for chromosome X variants [9]. The customized reference panel represented the merged panel of two reciprocally imputed reference panels: (1) 2,201 low‐coverage whole‐genome sequences samples from the HUNT study and (2) HRC v1.1 with 1,023 HUNT WGS samples removed before merging. We excluded imputed variants with Rsq < 0.3 resulting in over 24.9 million well‐imputed variants.”

**Procedure for costing contacts without an available DRG-weight**

For psychiatric and substance-abuse related contacts that did not have a registered DRG weight we used the average cost per policlinic consultation and one over-night inpatient stay for psychiatric and Interdisciplinary specialized drug treatment related contacts [10]. The cost of an inpatient stay was estimated by multiplying the average cost of one over-night inpatient stay with the number of days of inpatient care, and day-treatments were assumed to cost half of one over-night inpatient stay. For the years where the average costs were unavailable, we estimated the average cost for that year by KPI-adjusting the cost registered for the nearest available year. For contacts that did not have a registered DRG-weight, and that were not psychiatric or substance-abuse related contacts, we estimated the cost of this contact for a particular year based on the average cost registered for patients admitted with the same main diagnosis, or that underwent the same procedure during the same year.

**Power calculations**

We used the mRnd power calculator [11] to gauge the power of our effect estimates. We assumed an α=0.05, and an R^2^ = 0.027 based on the proportion of variation in BMI explained by the genetic variants reported by Locke et al. (2015) [12]. We used the non-stratified OLS estimates adjusted for study period, years of data available, birth year, and sex (β_GP_ = 5.8, β_specialist_ = 44.6) (Table 3), and variances (σ^2^ (BMI) = 19.47, σ^2^ (GP cost) = 32 836.6, and σ^2^ (specialist costs) =3.82*10^7^) calculated from our study sample. According to our calculations, we should be able to detect effect estimates of € 2.8 and above for GP costs, and € 97 and above for specialist costs (Figure S1) given our sample of 60 786 individuals.


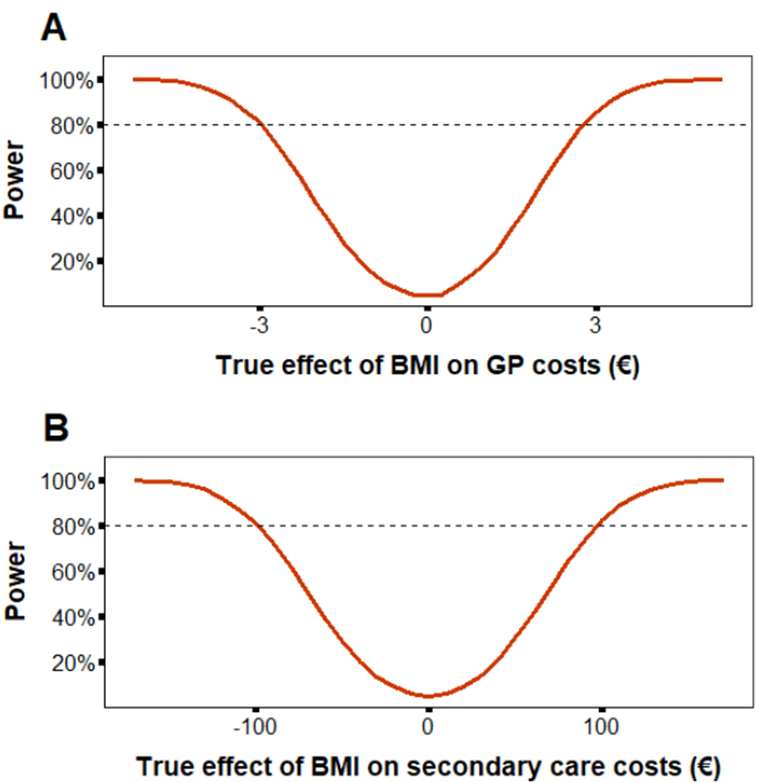


**Figure S1: Plot showing the amount of power to detect the true effect of BMI on A) GP costs, and B) specialist costs. The stippled line represents the 80% power threshold.**


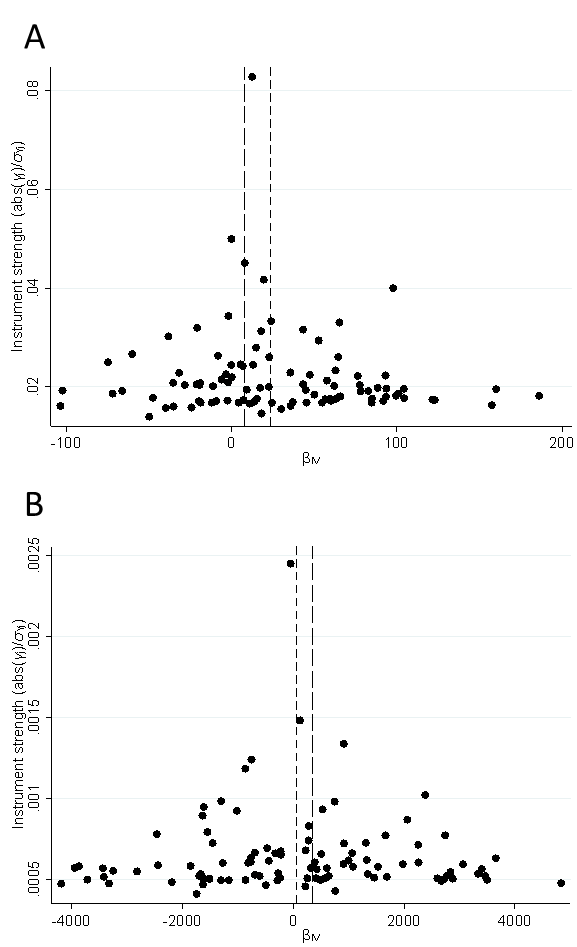


**Figure S2: Funnel plots for A) GP-costs and B) specialist costs: showing the estimated Wald ratios for each individual genetic variant plotted against the strength of the association between the genetic variant and BMI.**


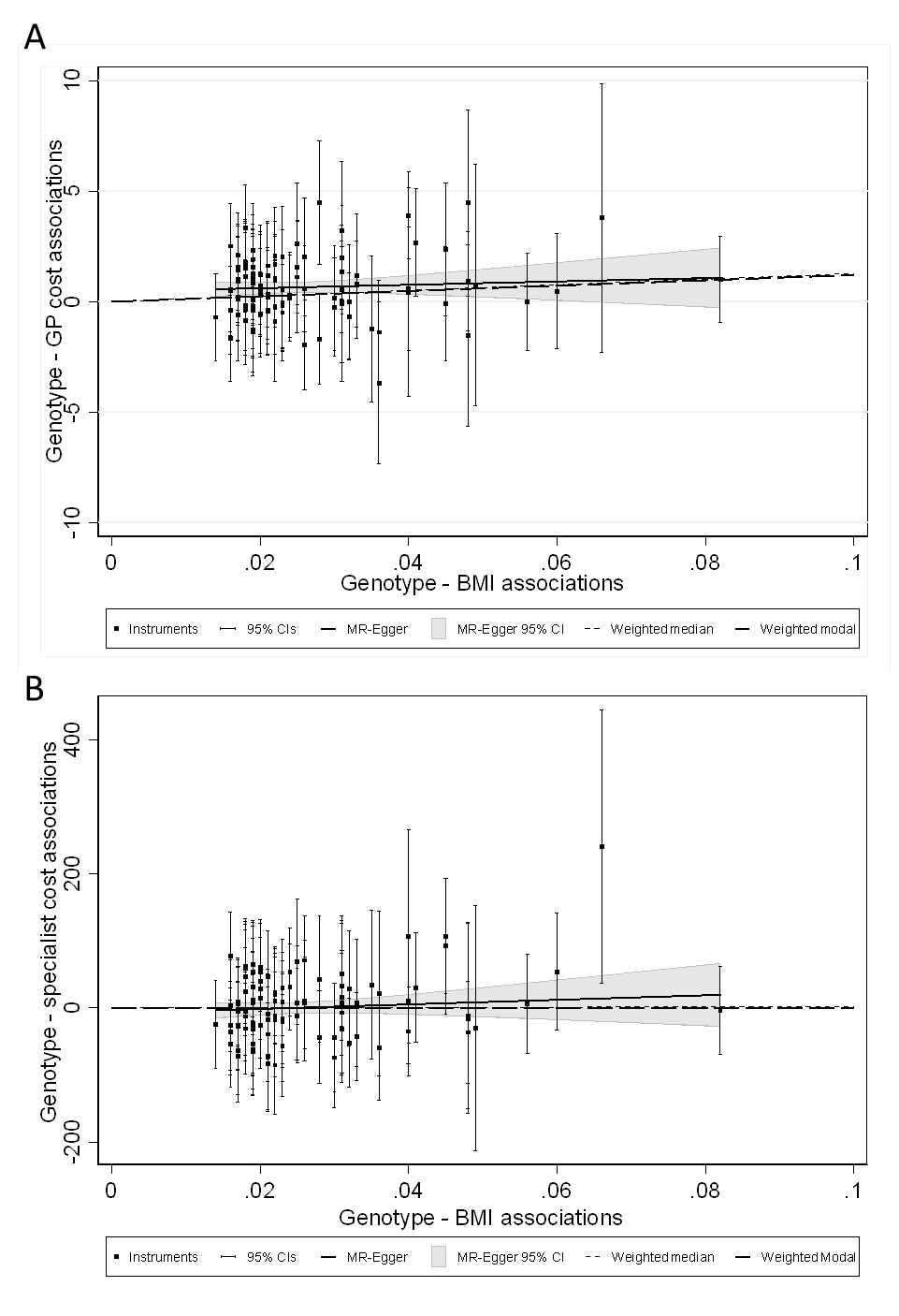


**Figure S3: Association between the BMI-related genetic variants and A) GP costs, and B) specialist costs, when using the IVW, MR-Egger, weighted-median, and weighted-modal estimators.**

**Figure S4: Forest plot showing the effect of each of the genetic variants on GP costs. The summary measures from the IVW and MR-Egger analyses are displayed at the bottom of the plot, and these are similar suggesting that bias from pleiotropy is likely to be small.**

**Figure S5: Forest plot showing the effect of each of the genetic variants on specialist costs. The summary measures from the IVW and MR-Egger analyses are displayed at the bottom of the plot, and these are similar suggesting that bias from pleiotropy is likely to be small.**


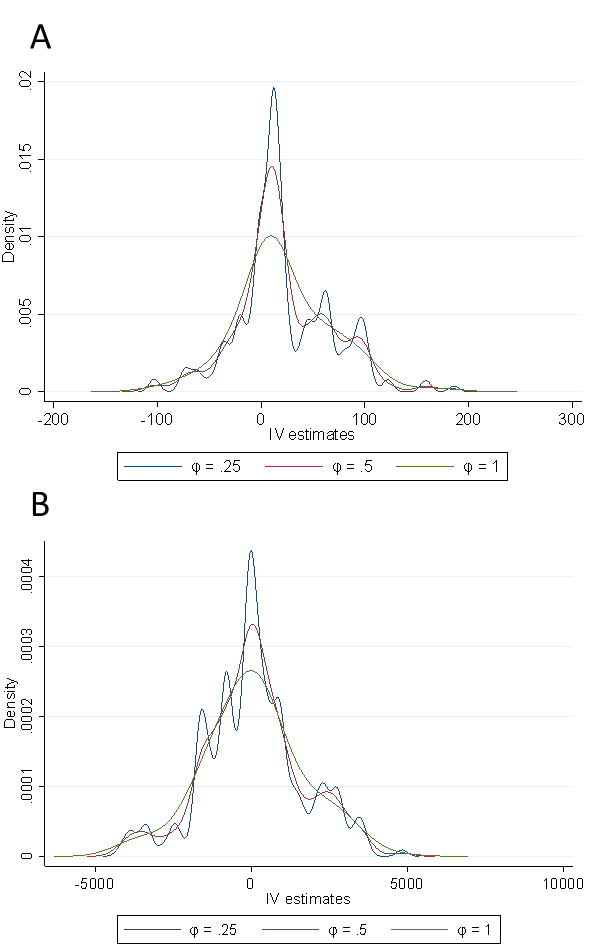


**Figure S6: Density plots for A) GP-costs and B) specialist costs with different bandwidth (smoothing parameter) values (**$\boldsymbol{\varphi}$**).**


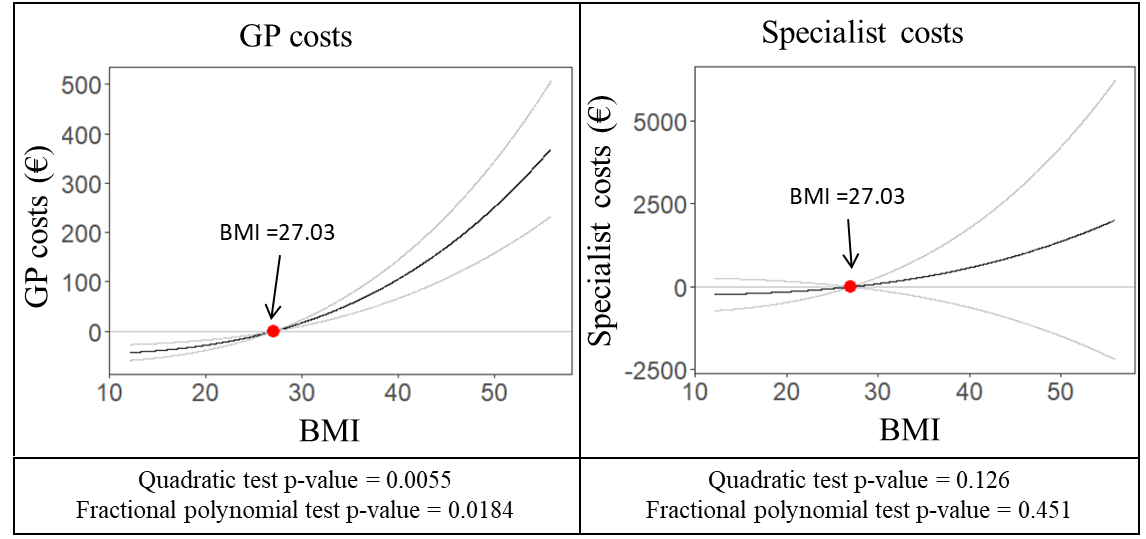


**Figure S7: Non-linear IV-models estimating GP costs (left) and specialist costs (right). The gradient at each point on the curve is the localised average causal effect. The red point indicates mean BMI, and the grey lines represent 95% confidence intervals.**

**Table S1: The regressed effect of each of the SNPs on primary care and secondary care costs. The values are adjusted for birthyear and sex.**

| SNPs | Primary care | | Specialist care | |
| --- | --- | --- | --- | --- |
|  | Beta coef. (SE) | 95% CI | Beta coef. (SE) | 95% CI |
| rs1000940 | 1.25 (1.05) | -0.82; 3.31 | -31.82 (35.45) | -101.3; 37.66 |
| rs10132280 | -0.05 (1.10) | -2.19; 2.10 | 30.47 (36.89) | -41.83; 102.77 |
| rs1016287 | -0.14 (1.07) | -2.25; 1.96 | -17.72 (36.14) | -88.55; 53.12 |
| rs10182181 | 1.34 (0.98) | -0.59; 3.27 | 16.37 (33.16) | -48.63; 81.37 |
| rs10733682 | 1.02 (0.99) | -0.91; 2.96 | 6.94 (33.22) | -58.17; 72.06 |
| rs10938397 | 3.91 (1.00) | 1.95; 5.87 | -34.76 (33.72) | -100.84; 31.33 |
| rs10968576 | 1.57 (1.07) | -0.52; 3.66 | -11.86 (35.95) | -82.33; 58.61 |
| rs11030104 | 2.67 (1.24) | 0.24; 5.09 | 30.54 (41.69) | -51.18; 112.25 |
| rs11057405 | 3.23 (1.58) | 0.12; 6.33 | 33.35 (53.35) | -71.22; 137.92 |
| rs11126666 | 0.19 (1.08) | -1.93; 2.32 | -82.99 (36.52) | -154.57; -11.41 |
| rs11165643 | 1.68 (0.99) | -0.26; 3.62 | 11.08 (33.29) | -54.17; 76.34 |
| rs11191560 | -0.08 (1.80) | -3.62; 3.46 | 7.80 (60.76) | -111.3; 126.9 |
| rs11583200 | -0.86 (1.01) | -2.83; 1.11 | -12.52 (33.88) | -78.92; 53.88 |
| rs1167827 | -0.57 (0.98) | -2.5; 1.35 | -25.57 (33.06) | -90.36; 39.23 |
| rs11688816 | 0.23 (1.00) | -1.72; 2.18 | -25.77 (33.53) | -91.48; 39.94 |
| rs11727676 | -3.69 (1.87) | -7.35; -0.03 | 21.62 (62.85) | -101.57; 144.82 |
| rs11847697 | 0.75 (2.78) | -4.70; 6.20 | -29.90 (93.60) | -213.37; 153.56 |
| rs12286929 | 1.04 (0.98) | -0.88; 2.96 | 23.27 (33.03) | -41.47; 88.02 |
| rs12401738 | -0.40 (1.01) | -2.37; 1.58 | -9.34 (33.95) | -75.89; 57.2 |
| rs12429545 | 1.17 (1.44) | -1.65; 3.98 | 7.29 (48.38) | -87.53; 102.11 |
| rs12446632 | 0.59 (1.43) | -2.21; 3.39 | 10.93 (48.04) | -83.23; 105.09 |
| rs12566985 | 0.16 (0.99) | -1.79; 2.11 | 54.13 (33.48) | -11.5; 119.76 |
| rs12885454 | 1.63 (1.03) | -0.38; 3.64 | 47.4 (34.55) | -20.33; 115.13 |
| rs12940622 | 1.68 (1.00) | -0.27; 3.64 | 24.16 (33.54) | -41.59; 89.9 |
| rs13021737 | 0.47 (1.33) | -2.13; 3.08 | 54.44 (44.77) | -33.32; 142.19 |
| rs13078960 | 0.16 (1.22) | -2.24; 2.56 | -43.79 (41.23) | -124.6; 37.02 |
| rs13107325 | 4.47 (2.15) | 0.26; 8.68 | -15.79 (72.28) | -157.46; 125.89 |
| rs13191362 | 4.48 (1.43) | 1.69; 7.28 | 42.75 (48.09) | -51.51; 137.02 |
| rs13201877 | 0.56 (1.37) | -2.12; 3.24 | -19.90 (46.01) | -110.07; 70.28 |
| rs1441264 | 1.14 (1.02) | -0.85; 3.14 | 62.33 (34.31) | -4.92; 129.57 |
| rs1460676 | 0.36 (1.37) | -2.34; 3.05 | 15.10 (46.26) | -75.58; 105.78 |
| rs1516725 | 2.37 (1.53) | -0.63; 5.38 | 92.65 (51.65) | -8.59; 193.89 |
| rs1528435 | 1.53 (1.02) | -0.46; 3.53 | -30.62 (34.34) | -97.94; 36.69 |
| rs1558902 | 1.01 (0.99) | -0.94; 2.95 | -4.08 (33.45) | -69.63; 61.48 |
| rs16851483 | -1.53 (2.10) | -5.66; 2.59 | -11.08 (70.87) | -149.98; 127.83 |
| rs16907751 | -1.24 (1.68) | -4.53; 2.04 | 34.89 (56.48) | -75.81; 145.59 |
| rs16951275 | 2.00 (1.19) | -0.33; 4.32 | 51.52 (40.00) | -26.89; 129.92 |
| rs17001654 | 0.00 (1.41) | -2.76; 2.76 | -6.94 (47.39) | -99.82; 85.95 |
| rs17024393 | 3.80 (3.10) | -2.26; 9.87 | 241.20 (104.21) | 36.94; 445.46 |
| rs17094222 | 1.12 (1.29) | -1.41; 3.66 | 7.80 (43.58) | -77.62; 93.21 |
| rs17203016 | 1.19 (1.20) | -1.16; 3.55 | -71.89 (40.45) | -151.17; 7.39 |
| rs17405819 | 0.95 (1.07) | -1.15; 3.05 | -16.93 (36.01) | -87.5; 53.64 |
| rs17724992 | 0.86 (1.13) | -1.36; 3.08 | -24.84 (38.10) | -99.51; 49.83 |
| rs1808579 | 2.09 (0.98) | 0.16; 4.02 | 9.84 (33.13) | -55.09; 74.78 |
| rs1928295 | 1.57 (0.99) | -0.36; 3.51 | -65.37 (33.24) | -130.53; -0.22 |
| rs2033529 | 2.31 (1.09) | 0.18; 4.44 | 32.00 (36.57) | -39.67; 103.67 |
| rs2033732 | 0.08 (1.13) | -2.13; 2.30 | -22.18 (38.11) | -96.87; 52.51 |
| rs205262 | 2.06 (1.12) | -0.13; 4.25 | -85.17 (37.63) | -158.93; -11.41 |
| rs2075650 | 2.03 (1.36) | -0.64; 4.71 | 10.92 (45.96) | -79.15; 101.00 |
| rs2080454 | 0.12 (0.98) | -1.80; 2.05 | -4.07 (33.12) | -68.98; 60.84 |
| rs2112347 | -1.95 (1.04) | -3.99; 0.08 | 7.13 (34.96) | -61.40; 75.67 |
| rs2121279 | 2.61 (1.41) | -0.16; 5.38 | 69.26 (47.61) | -24.06; 162.58 |
| rs2176040 | -0.70 (1.00) | -2.66; 1.27 | -24.48 (33.81) | -90.74; 41.79 |
| rs2176598 | 1.23 (1.15) | -1.04; 3.49 | 29.16 (38.85) | -46.99; 105.31 |
| rs2207139 | -0.09 (1.31) | -2.65; 2.46 | 107.12 (43.95) | 20.99; 193.25 |
| rs2245368 | -0.01 (1.31) | -2.57; 2.56 | 29.19 (44.12) | -57.28; 115.66 |
| rs2287019 | -1.38 (1.19) | -3.72; 0.96 | -59.07 (40.14) | -137.74; 19.61 |
| rs2365389 | 1.24 (0.99) | -0.71; 3.18 | 39.55 (33.43) | -25.98; 105.08 |
| rs2650492 | 0.36 (1.06) | -1.72; 2.44 | -39.01 (35.8) | -109.17; 31.16 |
| rs2820292 | 0.45 (1.00) | -1.50; 2.40 | 61.22 (33.5) | -4.45; 126.88 |
| rs2836754 | 0.48 (1.03) | -1.55; 2.50 | 3.44 (34.76) | -64.68; 71.57 |
| rs29941 | -0.17 (1.05) | -2.22; 1.89 | 46.75 (35.32) | -22.48; 115.98 |
| rs3101336 | 0.79 (0.99) | -1.16; 2.74 | -43.06 (33.48) | -108.69; 22.57 |
| rs3736485 | 1.79 (0.99) | -0.16; 3.74 | 59.97 (33.48) | -5.65; 125.59 |
| rs3810291 | -1.69 (1.05) | -3.74; 0.36 | -43.46 (35.21) | -112.48; 25.56 |
| rs3817334 | 0.59 (1.00) | -1.36; 2.55 | 71.23 (33.57) | 5.44; 137.02 |
| rs3849570 | -1.37 (1.02) | -3.37; 0.63 | -53.64 (34.38) | -121.02; 13.73 |
| rs3888190 | 0.55 (0.99) | -1.40; 2.50 | -31.72 (33.45) | -97.27; 33.83 |
| rs4256980 | -0.41 (1.03) | -2.43; 1.61 | -17.17 (34.65) | -85.09; 50.75 |
| rs4740619 | 3.35 (0.99) | 1.41; 5.28 | -4.98 (33.21) | -70.08; 60.12 |
| rs4771122 | -0.25 (1.14) | -2.49; 1.98 | -73.97 (38.37) | -149.17; 1.24 |
| rs4787491 | 2.52 (0.98) | 0.60; 4.43 | -35.16 (32.92) | -99.69; 29.37 |
| rs492400 | 0.57 (0.99) | -1.38; 2.52 | -53.23 (33.47) | -118.84; 12.38 |
| rs543874 | 0.93 (1.15) | -1.32; 3.18 | -36.31 (38.64) | -112.04; 39.42 |
| rs6091540 | 1.13 (1.08) | -0.98; 3.24 | 12.14 (36.24) | -58.88; 83.17 |
| rs6465468 | -0.60 (1.06) | -2.68; 1.47 | -71.28 (35.63) | -141.11; -1.45 |
| rs6477694 | 1.44 (1.01) | -0.55; 3.42 | -4.87 (34.17) | -71.85; 62.10 |
| rs6567160 | -0.01 (1.12) | -2.21; 2.19 | 6.73 (37.73) | -67.21; 80.67 |
| rs657452 | -0.08 (1.02) | -2.08; 1.92 | -16.00 (34.39) | -83.40; 51.40 |
| rs6804842 | -1.26 (0.99) | -3.20; 0.69 | 64.64 (33.47) | -0.96; 130.24 |
| rs7138803 | -0.67 (1.00) | -2.63; 1.29 | -51.83 (33.66) | -117.81; 14.15 |
| rs7141420 | 0.31 (0.98) | -1.61; 2.22 | 31.42 (32.94) | -33.14; 95.98 |
| rs7164727 | -0.34 (1.07) | -2.44; 1.75 | 62.93 (35.97) | -7.57; 133.44 |
| rs7239883 | -0.39 (1.01) | -2.36; 1.59 | -26.09 (33.9) | -92.53; 40.35 |
| rs7243357 | -0.88 (1.40) | -3.62; 1.86 | -10.99 (47.03) | -103.17; 81.20 |
| rs758747 | 2.03 (1.16) | -0.24; 4.29 | -56.2 (38.96) | -132.56; 20.17 |
| rs7599312 | -0.25 (1.09) | -2.39; 1.89 | 19.91 (36.71) | -52.04; 91.87 |
| rs7715256 | -1.66 (0.99) | -3.6; 0.29 | 77.34 (33.36) | 11.95; 142.73 |
| rs7899106 | 0.43 (2.41) | -4.29; 5.15 | 106.72 (81.08) | -52.19; 265.64 |
| rs7903146 | -0.49 (1.12) | -2.69; 1.71 | 8.85 (37.81) | -65.27; 82.96 |
| rs9374842 | -0.23 (1.13) | -2.44; 1.98 | 9.28 (38.01) | -65.22; 83.78 |
| rs9400239 | -0.38 (1.11) | -2.56; 1.80 | 54.64 (37.40) | -18.66; 127.95 |
| rs9540493 | 1.56 (0.99) | -0.38; 3.51 | -27.72 (33.41) | -87.20; 43.76 |
| rs9641123 | 1.92 (1.02) | -0.07; 3.91 | -61.88 (34.19) | -128.88; 5.13 |
| rs977747 | 0.93 (1.01) | -1.04; 2.90 | -63.27 (33.89) | -129.70; 3.16 |
| rs9914578 | 0.74 (1.18) | -1.57; 3.06 | 54.38 (39.70) | -23.43; 132.19 |
| rs9925964 | 0.95 (1.03) | -1.07; 2.96 | 53.88 (34.63) | -13.99; 121.75 |

**Table S2: Results from the one-sample 2SLS regressions for each type of healthcare cost and using three different instruments. As a sensitivity analysis, the estimates were adjusted for study period, years of data for which participants were alive and living in Norway, birth year, sex, educational level, marital status, smoking status, and urbanity.**

| Type of healthcare cost | Instrument | 2SLS First-stage Beta (SE) | 2SLS Second-stage  Beta € (SE) | F-stat. |
| --- | --- | --- | --- | --- |
| GP costs | Unweighted GRS | 0.1 (0.003)*** | 6.1 (1.181)*** | 1128.6 |
|  | Weighted GRS | 4.0 (0.104)*** | 5.2 (1.031)*** | 1491.6 |
|  | FTO & MC4R | 0.4 (0.025)*** &  0.3 (0.028)*** | 1.2 (2.163) | 168.3 |
| Specialist costs | Unweighted GRS | 0.1 (0.003)*** | -1.8 (40.467) | 1128.6 |
|  | Weighted GRS | 4.0 (0.104)*** | 15.4 (35.288) | 1491.6 |
|  | FTO & MC4R | 0.4 (0.025)*** &  0.3 (0.028)*** | 9.7 (73.589) | 168.3 |
| Total (GP + secondary care) costs | Unweighted GRS | 0.1 (0.003)*** | 4.3 (40.792) | 1128.6 |
|  | Weighted GRS | 4.0 (0.104)*** | 20.6 (35.572) | 1491.6 |
|  | FTO & MC4R | 0.4 (0.025)*** &  0.3 (0.028)*** | 10.8 (74.189) | 168.3 |

*** (p-value <0.001), ** (p-value <0.01), * (p-value<0.05), and **·** (p-value <0.1)

**Table S3: Beta coefficients and standard errors (SE) for the first- and second-stage results of the one-sample 2SLS regressions**^†^ **for each type of healthcare cost, using three different instruments. The estimates were adjusted for study period (HUNT 2 or HUNT 3), years of data participants were alive and living in the country during the cost estimation period (2009-2016), sex, birth year, and the first 10 genetic principal components.**

| Type of healthcare cost | Instrument | 2SLS First-stage Beta instrument (SE) | 2SLS Second-stage Beta BMI (SE) € |  |
| --- | --- | --- | --- | --- |
|  |  |  |  | F-stat. |
| General practitioner costs | Unweighted GRS | 0.1 (0.003)*** | 6.8 (1.186)*** | 1117.3 |
|  | Weighted GRS | 4.1 (0.105)*** | 5.7 (1.034)*** | 1478.2 |
|  | FTO &  MC4R | 0.4 (0.025)*** &  0.3 (0.028)*** | 1.8 (2.133) | 172.0 |
| Specialist costs | Unweighted GRS | 0.1 (0.003)*** | 1.1 (40.082) | 1117.3 |
|  | Weighted GRS | 4.0 (0.105)*** | 13.9 (34.940) | 1478.2 |
|  | FTO &  MC4R | 0.4 (0.025)*** &  0.3 (0.028)*** | -2.4 (71.793) | 172.0 |
| Total costs | Unweighted GRS | 0.1 (0.003)*** | 7.9 (40.411) | 1117.3 |
|  | Weighted GRS | 4.0 (0.105)*** | 19.5 (35.228) | 1478.2 |
|  | FTO &  MC4R | 0.4 (0.025)*** &  0.3 (0.028)*** | -0.5 (72.392) | 172.0 |

*** (p-value <0.001), ** (p-value <0.01), * (p-value<0.05), and **·** (p-value <0.1)

**Table S4: The estimated effect of BMI on GP-costs and specialist costs for both sexes, and for males and females, with 0 or 96 invalid instruments.**

|  | Males and females | | Males | | Females | |
| --- | --- | --- | --- | --- | --- | --- |
|  | Est. Coef  [0 invalid instruments] | Est. Coef.  [96 invalid instruments] | Est. Coef  [0 invalid instruments] | Est. Coef.  [96 invalid instruments] | Est. Coef  [0 invalid instruments] | Est. Coef.  [96 invalid instruments] |
| GP costs | 6.46 | 5.20 | 6.59 | 10.5 | 6.28 | 1.12 |
| Specialist costs | 54.51 | 84.52 | 125.86 | 2.21x10^11^ | 4.74 | 8.10x10^8^ |

**Table S5: Sex-specific, first- and second-stage results of the one-sample 2SLS regressions for each type of healthcare cost, using three different instruments. The estimates were adjusted for study period (HUNT 2 or HUNT 3), years of data participants were alive and living in the country during the cost estimation period (2009-2016), and birth year.**

| Type of healthcare cost |  | Instrument | 2SLS First-stage  Beta (SE) | 2SLS Second-stage  Beta (SE) | F-stat. |
| --- | --- | --- | --- | --- | --- |
|  | Sex |  |  |  |  |
| GP | Male | Unweighted GRS | 0.1 (0.003)*** | 7.6 (1.790)*** | 591.3 |
|  |  | Weighted GRS | 3.6 (0.133)*** | 6.4 (1.589)*** | 754.3 |
|  |  | FTO &  MC4R | 0.4 (0.032)*** &  0.2 (0.036)*** | -3.1 (3.528)**·** | 79.0 |
|  | Female | Unweighted GRS | 0.1 (0.004)*** | 6.1 (2.717)*** | 567.5 |
|  |  | Weighted GRS | 4.3 (0.154)*** | 5.4 (1.328)*** | 777.1 |
|  |  | FTO &  MC4R | 0.4 (0.038)***&  0.4 (0.043)*** | 4.7 (2.629) | 97.5 |
| Specialist | Male | Unweighted GRS | 0.1 (0.003)*** | 54.2 (68.859) | 591.3 |
|  |  | Weighted GRS | 3.6 (0.133)*** | 92.9 (61.159) | 754.3 |
|  |  | FTO &  MC4R | 0.4 (0.032)*** &  0.2 (0.036)*** | 136.0 (132.365) | 79.0 |
|  | Female | Unweighted GRS | 0.1 (0.004)*** | -32.6 (47.733) | 567.5 |
|  |  | Weighted GRS | 4.3 (0.154)*** | -39.5 (40.938) | 777.1 |
|  |  | FTO &  MC4R | 0.4 (0.038)***&  0.4 (0.043)*** | -80.9 (81.286) | 97.5 |
| Total | Male | Unweighted GRS | 0.1 (0.003)*** | 61.8 (69.320) | 591.3 |
|  |  | Weighted GRS | 3.6 (0.133)*** | 99.3 (61.567) | 175.3 |
|  |  | FTO &  MC4R | 0.4 (0.032)*** &  0.2 (0.036)*** | 132.9 (133.209) | 79.0 |
|  | Female | Unweighted GRS | 0.1 (0.004)*** | -26.4 (48.195) | 567.5 |
|  |  | Weighted GRS | 4.3 (0.154)*** | -34.1 (41.335) | 777.1 |
|  |  | FTO &  MC4R | 0.4 (0.038)***&  0.4 (0.043)*** | -76.2 (82.077) | 97.5 |

*** (p-value <0.001), ** (p-value <0.01), * (p-value<0.05), and **·** (p-value <0.1)

**Table S6: Sex-specific, first- and second-stage results of the one-sample 2SLS regressions for specialist costs by type of healthcare provider, using three different instruments. The estimates were adjusted for study period (HUNT 2 or HUNT 3), years of data participants were alive and living in the country during the cost estimation period (2009-2016), and birth year.**

| Specialist healthcare provider | Sex | Instrument | 2SLS First-stage Beta (SE) | | 2SLS Second-stage  Beta (SE) | | F-stat | |
| --- | --- | --- | --- | --- | --- | --- | --- | --- |
| Somatic hospital | Both | Unweighted GRS | 0.1 (0.003)*** | | 21.2 (33.086) | | 1119.4 | |
|  |  | Weighted GRS | 4.0 (0.105)*** | | 19.5 (25.863) | | 1481.4 | |
|  |  | FTO &  MC4R | 0.4 (0.025)*** &  0.3 (0.028)*** | | 4.7 (59.220) | | 172.6 | |
|  | Male | Unweighted GRS | 0.1 (0.003)*** | | 92.1 (61.369) | | 591.3 | |
|  |  | Weighted GRS | 3.7 (0.133)*** | | 96.8 (54.495)· | | 754.3 | |
|  |  | FTO &  MC4R | 0.4 (0.032)*** &  0.2 (0.036)*** | | 127.4 (117.931) | | 79.0 | |
|  | Female | Unweighted GRS | 0.1 (0.004)*** | | -29.2 (35.708) | | 567.5 | |
|  |  | Weighted GRS | 4.3 (0.154)*** | | -41.0 (30.647) | | 777.1 | |
|  |  | FTO &  MC4R | 0.4 (0.038)*** &  0.4 (0.043)*** | | -64.8 (60.823) | | 97.5 | |
| Contracted provider of somatic and psychological healthcare | Both | Unweighted GRS | 0.1 (0.003)*** | | -3.9 (4.350) | | 1119.4 | |
|  |  | Weighted GRS | 4.0 (0.105)*** | | -1.2 (3.8794) | | 1481.4 | |
|  |  | FTO &  MC4R | 0.4 (0.025)*** &  0.3 (0.028)*** | | 3.0 (7.782) | | 172.6 | |
|  | Male | Unweighted GRS | 0.1 (0.003)*** | | 2.3 (5.593) | | 591.3 | |
|  |  | Weighted GRS | 3.7 (0.133)*** | | 3.7 (4.966) | | 754.3 | |
|  |  | FTO &  MC4R | 0.4 (0.032)*** &  0.2 (0.036)*** | | 9.7 (10.749) | | 79.0 | |
|  | Female | Unweighted GRS | 0.1 (0.004)*** | | -8.4 (6.342) | | 567.5 | |
|  |  | Weighted GRS | 4.3 (0.154)*** | | -4.4 (5.431) | | 777.1 | |
|  |  | FTO &  MC4R | 0.4 (0.038)*** &  0.4 (0.043)*** | | -0.2 (10.745) | | 97.5 | |
| Psychiatric care | Both | Unweighted GRS | 0.1 (0.003)*** | | 1.4 (16.370) | | 1119.4 | |
|  |  | Weighted GRS | 4.0 (0.105)*** | | 4.1 (14.280) | | 1481.4 | |
|  |  | FTO &  MC4R | 0.4 (0.025)*** &  0.3 (0.028)*** | | 15.6 (29.298) | | 172.6 | |
|  | Male | Unweighted GRS | 0.1 (0.003)*** | | -11.8 (20.350) | | 591.3 | |
|  |  | Weighted GRS | 3.7 (0.133)*** | | 3.9 (18.061) | | 754.3 | |
|  |  | FTO &  MC4R | 0.4 (0.032)*** &  0.2 (0.036)*** | | 28.7 (39.102) | | 79.0 | |
|  | Female | Unweighted GRS | 0.1 (0.004)*** | | 11.4 (24.186) | | 567.5 | |
|  |  | Weighted GRS | 4.3 (0.154)*** | | 9.3 (20.732) | | 777.1 | |
|  |  | FTO &  MC4R | 0.4 (0.038)*** &  0.4 (0.043)*** | | 4.8 (41.025) | | 97.5 | |
| Interdisciplinary Specialized Drug Treatment | Both | Unweighted GRS | 0.1 (0.003)*** | -5.1 (7.304) | | 1119.4 | |  |
|  |  | Weighted GRS | 4.0 (0.105)*** | | -2.1 (6.370) | | 1481.4 | |
|  |  | FTO &  MC4R | 0.4 (0.025)*** &  0.3 (0.028)*** | | -20.6 (13.114) | | 172.6 | |
|  | Male | Unweighted GRS | 0.1 (0.003)*** | | -11.8 (13.625) | | 591.3 | |
|  |  | Weighted GRS | 3.7 (0.133)*** | | -4.4 (12.093) | | 754.3 | |
|  |  | FTO &  MC4R | 0.4 (0.032)*** &  0.2 (0.036)*** | | -21.3 (26.188) | | 79.0 | |
|  | Female | Unweighted GRS | 0.1 (0.004)*** | | 0.5 (7.821) | | 567.5 | |
|  |  | Weighted GRS | 4.3 (0.154)*** | | -0.6 (6.705) | | 777.1 | |
|  |  | FTO &  MC4R | 0.4 (0.038)*** &  0.4 (0.043)*** | | -20.5 (13.362) | | 97.5 | |

*** (p-value <0.001), ** (p-value <0.01), * (p-value<0.05), and **·** (p-value <0.1)

**Table S7: First- and second-stage results of the one-sample 2SLS regressions for GP costs with outliers excluded, using three different instruments. The estimates were adjusted for study period (HUNT 2 or HUNT 3), years of data participants were alive and living in the country during the cost estimation period (2009-2016), sex, and birth year.**

|  | Instrument | 2SLS First Stage  Beta (SE) | 2SLS Second-stage  Beta (SE) |  |
| --- | --- | --- | --- | --- |
| Outliers excluded |  |  |  | F-stat. |
| rs4740619 | Unweighted GRS | 0.1 (0.003)*** | 6.2 (1.181)*** | 1113.2 |
|  | Weighted GRS | 4.1 (0.105)*** | 5.4 (1.029)*** | 1476.1 |
| rs13191362 | Unweighted GRS | 0.1 (0.003)*** | 6.4 (1.182)*** | 1112.5 |
|  | Weighted GRS | 4.0 (0.105)*** | 5.4 (1.030)*** | 1472.1 |
| rs4787491 | Unweighted GRS | 0.1 (0.003)*** | 6.3 (1.182)*** | 1111.7 |
|  | Weighted GRS | 4.0 (0.105)*** | 5.5 (1.030)*** | 1473.6 |
| rs4740619, rs13191362, & rs4787491 | Unweighted GRS | 0.1 (0.003)*** | 5.7 (1.193)*** | 1091.8 |
|  | Weighted GRS | 4.0 (0.106)*** | 5.0 (1.037)*** | 1453.0 |

*** (p-value <0.001), ** (p-value <0.01), * (p-value<0.05), and **·** (p-value <0.1)

**Table S8: First- and second-stage results of the one-sample 2SLS regressions for specialist costs with the outlier excluded, using three different instruments. The estimates were adjusted for study period (HUNT 2 or HUNT 3), years of data participants were alive and living in the country during the cost estimation period (2009-2016), sex, and birth year.**

| Outliers excluded | Instrument | 2SLS First Stage  Beta (SE) | 2SLS Second-stage  Beta (SE) | F-stat. |
| --- | --- | --- | --- | --- |
| rs7715256 | Unweighted GRS | 0.1 (<0.003)*** | -8.3 (40.195) | 1114.6 |
|  | Weighted GRS | 4.0 (0.105)*** | 9.4 (35.033) | 1474.5 |

**Table S9: First- and second-stage results of the one-sample 2SLS regressions for GP- and specialist costs within families (N=29 199), using three different instruments. The estimates were adjusted for study period (HUNT 2 or HUNT 3), years of data participants were alive and living in the country during the cost estimation period (2009-2016), sex, and birth year.**

| Type of healthcare cost | Instrument | 2SLS First-stage | 2SLS Second-stage |
| --- | --- | --- | --- |
|  |  | Beta (SE) | Beta (SE) € |
| GP | Unweighted GRS | 0.1 (0.006)*** | 2.7 (2.458) |
|  | Weighted GRS | 4.3 (0.235)*** | 1.7 (2.185) |
|  | FTO &  MC4R | 0.4 (0.056)*** &  0.4 (0.064)*** | 0.8 (3.970) |
| Specialist | Unweighted GRS | 0.1 (0.006)*** | -65.6 (91.458) |
|  | Weighted GRS | 4.3 (0.235)*** | -83.7 (81.239) |
|  | FTO &  MC4R | 0.4 (0.056)*** &  0.4 (0.064)*** | -155.9 (147.701) |

*** (p-value <0.001), ** (p-value <0.01), * (p-value<0.05), and **·** (p-value <0.1)





**Figure S8: Simple overview of the number of contacts made by males and females to GPs (left) and somatic hospital care (right) for different diagnoses, during 2012 in Norway, based on aggregated data from Statistics Norway [13,14] Females generally had higher number of contacts, but males had more GP consultations due to heart disease, diabetes, cancer, administrative contacts, and accidents and injuries, and had more somatic hospital visits due to injuries and poisonings, infectious and parasitic diseases, diseases of the respiratory system, diseases of the ear, and diseases of the circulatory system.**

# **References**

1. Jun, G., Flickinger, M., Hetrick, K. N., Romm, J. M., Doheny, K. F., Abecasis, G. R., . . . Kang, H. M. (2012). **Detecting and estimating contamination of human DNA samples in sequencing and array‐based genotype data.** Am J Hum Genet, 91 (5), 839‐848. doi:10.1016/j.ajhg.2012.09.004.

2. Guo, Y., He, J., Zhao, S., Wu, H., Zhong, X., Sheng, Q., . . . Long, J. (2014). **Illumina human exome genotyping array clustering and quality control.** Nat Protoc, 9 (11), 2643‐2662.

3. Dunham, I., Kundaje, A., Aldred, S. F., Collins, P. J., Davis, C. A., Doyle, F., . . . Lochovsky, L. (2012). **Anintegrated encyclopedia of DNA elements in the human genome.** Nature, 489 (7414), 57‐74. doi:nature11247 [pii] 10.1038/nature11247.

4. Li, J. Z., Absher, D. M., Tang, H., Southwick, A. M., Casto, A. M., Ramachandran, S., . . . Myers, R. M. (2008). **Worldwide human relationships inferred from genome‐wide patterns of variation.** Science, 319 (5866), 1100‐1104. doi:10.1126/science.1153717.

5. Wang, C., Zhan, X., Bragg‐Gresham, J., Kang, H. M., Stambolian, D., Chew, E. Y., . . . Abecasis, G. R. (2014). **Ancestry estimation and control of population stratification for sequence‐based association studies.** Nat Genet, 46 (4), 409‐415. doi:10.1038/ng.2924.

6. Chang, C. C., Chow, C. C., Tellier, L. C., Vattikuti, S., Purcell, S. M., & Lee, J. J. (2015). **Second‐generation PLINK: rising to the challenge of larger and richer datasets.** Gigascience, 4 , 7. doi:10.1186/s13742‐015‐0047‐8.

7. Loh, P.‐R., Danecek, P., Palamara, P. F., Fuchsberger, C., Reshef, Y. A., Finucane, H. K., . . . Price, A. L. (2016). **Reference‐based phasing using the Haplotype Reference Consortium panel.** bioRxiv . doi: <http://dx.doi.org/10.1101/052308>.

8. Das, S., Forer, L., Schonherr, S., Sidore, C., Locke, A. E., Kwong, A., . . . Fuchsberger, C. (2016). **Next‐generation genotype imputation service and methods.** Nat Genet .doi:10.1038/ng.3656.

9. McCarthy, S., Das, S., Kretzschmar, W., Delaneau, O., Wood, A. R., Teumer, A., . . . Haplotype Reference, C. (2016**). A reference panel of 64,976 haplotypes for genotype imputation.** Nat Genet, 48 (10), 1279‐1283. doi:10.1038/ng.3643.

10. Norwegian Directorate of Health. (2018). Hovedresultater Samdata Spesialisthelsetjenesten 2013-2017.

11. Brion, M.-J.A., Shakhbazov, K., & Visscher, P.M. (2013). **Calculating statistical power in Mendelian randomization studies.** International Journal of Epidemiology, 42, 1497-1501.DOI: 10.1093/ije/dyt179.

12. Locke, A.E., Kahali, B., Berndt, S.I., Justice, A.E., Pers, T.H., Day, F.R., et al. (2015). **Genetic studies of body mass index yield new insights for obesity biology.** Nature, 518, 197-206.DOI: 10.1038/nature14177.

13. Statistics Norway. Tabell 10141: Konsultasjoner hos fastlegen, etter alder, kjønn og diagnose 2012 - 2018.

14. Statistics Norway. Tabell 10261: Pasienter, behandlinger og liggedager ved somatiske sykehus, etter kjønn, alder og diagnose (F) 2012 - 2018.
